# A deep residual 1D-CNN with self-attention for fraud transaction detection in virtual economies

**DOI:** 10.1038/s41598-026-37032-w

**Published:** 2026-02-12

**Authors:** Kamel K. Mohammed, Asmaa S. Abdo, Ashraf Darwish, Aboul Ella Hassanien

**Affiliations:** 1 Center for Virus Research and Studies, Al-Azhar University, Cairo, 11754 Egypt; 2 Information Systems Department, Faculty of Computers and Artificial Intelligence, University of Sadat City, Sadat City, Menoufia 32897 Egypt; 3 Faculty of Science, Capital University, Cairo, Egypt; 4 Faculty of Computers and Artificial Intelligence, Obour University for Science and Technology, Cairo, Egypt; 5 Faculty of Computer and Artificial Intelligence, Cairo University, Cairo, Egypt; 6 Scientific Research School of Egypt (SRSEG), Cairo, Egypt

**Keywords:** Metaverse, Virtual economies, Financial transactions, Fraud detection, Risk classification, Convolutional neural network (CNN), Self-attention mechanism, Computer science, Information technology

## Abstract

As virtual economies in the metaverse continue to grow, the need for real-time risk assessment in financial transactions has become critical. Traditional fraud detection systems often face challenges in keeping pace with the complexity and speed of metaverse data. To address this, we introduce a real-time anomaly detection and risk classification model designed specifically for metaverse transactions. The model is based on a one-dimensional convolutional neural network (1D-CNN) enhanced with residual connections and a self-attention mechanism, allowing it to focus on the most relevant features of each transaction for improved risk classification. We trained the model on benchmark metaverse financial datasets from Kaggle, achieving excellent results in accuracy, sensitivity, and specificity when classifying transactions into three risk levels—low, moderate, and high. To validate its robustness, we also tested it on the widely used Credit Card Fraud Detection dataset, where it maintained strong performance. However, we acknowledge that perfect scores can sometimes indicate overly clean or predictable data. To address this, we conducted an ablation study by introducing controlled noise into the dataset, evaluating the model’s ability to handle uncertainty and imperfections in real-world scenarios. To enhance interpretability, we analyzed feature importance across several CNN-based variations and assessed performance using confusion matrices, ROC curves, and t-SNE visualizations, which confirmed clear separation of risk levels in high-dimensional space. Further comparisons with other machine learning and deep learning models demonstrate the confidence and effectiveness of the proposed 1D-CNN architecture for financial fraud detection in the metaverse.

## Introduction

The metaverse concept refers to digital three-dimensional environments driven by virtual and augmented reality technologies^1^. The metaverse has the potential to become the next generation of the internet, possibly defining Web 3.0 or at least becoming an integral part of it. It represents an immersive and persistent convergence of the physical and digital worlds where people can connect, interact, and collaborate. The metaverse as an extension of real life enables users to participate in a variety of activities online^2^. The financial sector is rapidly adopting the metaverse, with banks recognizing the transformative potential of virtual reality in client services and financial transactions. Traditional fraud detection and anomaly classification systems do not address the high-dimensional, imbalanced, and sequential nature of metaverse transaction data. Specific challenges include lack of labelled data with varied risk annotations, masked user identities and location spoofing, and multi-class risk profiling in real-time. In recent years, the financial sector has transitioned from traditional methods to digital finance to enhance technological advancements. The metaverse greatly increases access to economic benefits. It facilitates seamless and secure transactions, improves customer communication, and contributes to achieving carbon net-zero goals. Also, provides a unique opportunity for bankers to improve their operations and remain a step ahead of the competition^3^. The traditional industrial physical economy has evolved into an informational virtual economy^4,5^. It can enhance access to financial services and provide financial education^6^.

Research on the adoption of metaverse technologies has mainly focused on organizational contexts^1^. Existing models often rely on binary classification and are not tuned to handle real-time, multi-class risk levels in a metaverse environment. Three core technologies driving the benefits of the metaverse in financial transactions are blockchain, digital assets, and smart contracts. Blockchain is a decentralized network where data blocks are interconnected. It plays a crucial role in facilitating financial transactions within virtual environments. Blockchain technology is the foundation for cryptocurrency operations. Efforts are being made to create a new financial ecosystem using blockchain technology in the financial sector^7^. The metaverse integrates previous technological developments with its unique technologies, making finance more accessible and reliable. The transformation of finance spans from traditional finance to digital finance, decentralized finance, and now, metaverse finance. An overview of companies from chosen countries that have developed in the metaverse reveals that firms in information technology sector are expected to have made the most investments with 17% of companies in this sector already investing^8^. The education sector follows closely, with 12% of businesses pursuing this opportunity. The top finance companies in the finance market using metaverse are presented in^2^. It lists various companies and their revenues. Leading finance companies are also using the metaverse with revenues ranging from US$130 billion for JPMorgan Chase to US$10 million for IBK Group. It represented countries include the USA, UK, France, South Korea, British Columbia, and Kuwait^2^.

The potential for artificial intelligence (AI) within the metaverse is vast. AI involves techniques such as machine and deep learning. Deep learning includes convolutional neural networks (CNNs). A specific type of CNN is the one-Dimensional convolutional neural network (1D-CNN), is particularly effective for analyzing sequential data such as time series or text^9^. AI can enhance the metaverse by improving accessibility and offering advanced security and privacy features. By utilizing AI algorithms to analyze and learn from blockchain data, significant advancements can be achieved^8^. AI can identify and avoid illegal activities on blockchain networks^9^. In recent years, the deep neural networks have achieved success in terms of both their advancement as well as their performance. The most significant between these is CNNs. Modern CNN architectures still hold fundamental features of the original design such as convolutional and pooling layers. CNNs are trained in supervised models with the backpropagation algorithm and they best at extracting hierarchical feature representations from raw data.

This paper presents a new deep learning model designed to quickly detect and classify risks in metaverse financial transactions. What makes this model different is that it combines 1D Convolutional Neural Networks (1D-CNN) with ResNet connections and a self-attention mechanism. This combination helps the model focus on the most important parts of each transaction and understand the order of events better.

The model works in three main phases, which are data preprocessing, data separation (training, validation and testing), and classifying the risk level phase. In the first step, the data is cleaned, balancing and encoding (to turn text into numbers the model can understand). For example, features like “Age Group” or “Location Region” are converted into numbers using methods like One-Hot Encoding or Sequential Encoding. The second phase the pre-proceed datasets, is divided into three categories including training, validation and testing. The Last phase is classification of the fraud transaction levels.

To make sure the model isn’t just memorizing clean data, we also tested it with noisy data to see how well it performs in real-world situations. We even used t-SNE to visualize how the model separates different risk levels, and the results showed clear differences between low, medium, and high-risk transactions.

Overall, this model offers a smart and reliable way to help metaverse platforms and financial institutions monitor transaction risks in real time.

This paper presents several key contributions to the field of risk classification in metaverse financial transactions including:

- A deep learning model developed for real-time anomaly detection in virtual financial systems within metaverse environments.
- An enhanced 1D-CNN architecture was proposed that integrates residual connections and an attention mechanism to improve risk classification performance.
- Handling both categorical and numerical features using categorical feature encoding techniques.
- The application of Random Over-Sampling (ROS) was used to address class imbalance, improving the representation of minority classes and enhancing classification reliability.
- Stratified 5-Fold Cross-Validation performance and statistical analysis.
- An ablation study was conducted to assess the individual and combined effects of CNN layers, residual connections, and attention mechanisms on model performance and training efficiency.
- Comprehensive evaluation of model performance through confusion matrix analysis, ROC curve analysis, and t-SNE visualization, confirming the model’s ability to effectively classify transactions into low, moderate, and high-risk levels.
- Feature importance analysis conducted across several CNN-based variations to identify critical features contributing to risk classification.
- Ablation study and noise robustness evaluation to assess the model’s stability and performance under real-world conditions with controlled noise.
- Comparative evaluation of the proposed model against other machine and deep learning models on the metaverse financial transaction dataset, demonstrating the effectiveness of the proposed architecture for fraud detection.
- Evaluation of classification performance before and after oversampling, confirming the model’s reliability and robustness.
- Comparative experiment on the Credit Card Fraud Detection dataset obtained from Kaggle, assessing the classification performance of the proposed 1D-CNN architecture.

The remainder of this paper is structured as follows. Section “Related work” provides a review of related work and discusses the research gaps. Section “Materials and methods” details the materials and methods employed in the research. Section “Dataset overview and characteristics” presents the dataset characteristics. Section “Proposed financial transactions classification model in metaverse” introduces the proposed model, followed by Sect. “Experimental results and evaluations”, which describes the experimental setup and presents an evaluation of the results. Finally, Sects. “Discussion” and “Conclusion and future work” present the discussion, highlight the key results, and conclude the research.

## Related work

Research on the adoption of the metaverse has been primarily focused on gaming, education, and sports with limited attention given to financial transactions. This section reviews relevant research papers on financial transactions within the metaverse. It also highlighting key research efforts, methodologies, and challenges. Vakiti et al.^10^ provides a comprehensive review and analysis of the current state of fintech in metaverse banking. Ooi et al.^11^ investigate the effect of the metaverse on the banking sector. Ritterbusch et al.^4^ analyze scientific definitions and descriptions of the term “metaverse” to provide a comprehensive understanding of this emerging concept. It discusses the social, economic, and technical implications of the metaverse, highlighting challenges and research opportunities, such as the need for standards and infrastructure.

Sitnikov et al.^12^ examine the scenarios of financial and legal development in metaverses through the use of digital currencies. The research analyzes the financial and legal policies of different authorities. Lyoussi et al.^6^ examine the historical development of the metaverse. It discusses its impact on the banking industry along with the associated opportunities and risks. Mozumder et al.^2^ highlight the potential applications of the metaverse in the finance industry. It emphasizes the financial opportunities that the metaverse offers, both for individuals and companies. The authors describe how financial institutions are leveraging virtual reality and augmented reality to enhance customer experience and engagement. The research also provides insights into the applications, challenges, and potential of the metaverse in the finance industry. Hong et al.^7^ examines and analyzes the diverse applications and utilization of blockchain technology for various types of virtual assets. It analyzes the utilization of blockchain for different virtual asset applications like cryptocurrency, decentralized finance, central bank digital currency, non-fungible tokens, and metaverses. Nguyen et al.^1^ investigate the adoption of metaverse banking services. The authors are developing a comprehensive model. It incorporates metaverse trust and metaverse financial resources to account for complexity within the metaverse environment. The limitation of the model is its focus on Vietnam country, which may limit the generalizability of the results. Hasan et al.^13^ provides a comprehensive evaluation of fraud detection in Bitcoin transactions. Chung et al.^14^ have explored deeper architecture with residual connections for anomaly detection. It developed a parallel CNN-LSTM model with residual blocks and attention for anomaly detection. Iqbal et al.^15^ investigates the potential of the Metaverse in the financial industry. It identifies use cases, value propositions, and challenges. Yu et al.^16^ proposes a novel hybrid model for financial fraud detection The key contributions include the development of a quantum-enhanced Deep Belief Network. It achieves a precision of 88.7% and recalls of 86.5% outperforming traditional methods. The model establishes robustness in detecting complex fraud patterns by fusing multi-dimensional features, reducing economic losses and deployment costs for financial institutions. Wu et al.^17^ propose an enhanced CNN-LSTM-Attention model optimized by the Sparrow Search Algorithm for detecting corporate accounting fraud. The research advances fraud detection by combining deep learning with bio-inspired optimization. It presents a more accurate and efficient solution for financial applications. Luo et al.^18^ proposes a hybrid CNN-BiGRU-AM model integrated with anomaly detection for nonlinear stock price prediction. It combines CNN for local feature extraction, BiGRU for bidirectional temporal modelling, and an attention mechanism to weight critical features, while an auto-encoder filters anomalies. The model achieves performance such as R^2^ = 0.9903, RMSE = 22.027, and Sharpe Ratio = 0.65 over Shanghai Composite Index data from 1991 to 2020. Prayitno et al.^19^ contribute to the emerging field of metaverse blockchain transaction analysis. It presents a comprehensive evaluation of six clustering algorithms such as K-Means, DBSCAN, GMM, Mean Shift, Spectral Clustering, and Birch on data. The results show that K-Means is the higher clustering approach for this domain. It achieves the highest performance scores (Silhouette Score: 0.4702, Calinski-Harabasz Index: 151946.29, Davies-Bouldin Index: 0.6600), while DBSCAN and Spectral Clustering presented less effective results, and GMM and Birch showed intermediate performance. Li et al.^20^ employing machine learning models for anomaly detection in Metaverse financial transactions. The research also establishes a comprehensive risk scoring model to enhance fraud detection. It highlights the effectiveness of Random Forest and XGBoost models, which show high accuracy and low error rates. The limitations in this research include issues related to imbalance data that could impact model predictions. Srinivasan et al.^21^ discuss blockchain financial transactions in metaverse. It focuses on risk analysis, and anomaly detection. The research includes the development of machine learning models such as Logistic Regression, Random Forest, and K-Means clustering to detect transactional anomalies and assess risk. It utilizes a dataset of 78,600 transactions. The results highlight the effectiveness of anomaly detection in identifying fraudulent activities. Jabeen et al.^22^ is a hybrid Convolutional Neural Network (CNN) and Long Short-Term Memory (LSTM) architecture designed for credit card fraud detection. It leverages CNNs to extract spatial features and LSTMs to capture temporal patterns in transaction sequences, thereby learning both individual transaction details and sequential spending behavior. The CLST model addresses severe class imbalance through the Synthetic Minority Oversampling Technique (SMOTE) during preprocessing and achieves high performance (AUC-ROC of ~ 0.9995 and accuracy of ~ 99.98%) on a public credit card fraud dataset through extensive hyperparameter tuning.

Despite these advancements, most existing studies either focus on traditional financial systems or general metaverse applications, with few addressing the unique characteristics of financial transactions within the metaverse. There is a lack of models specifically designed to handle the sequential, categorical, and often noisy nature of metaverse transaction data. This paper addresses that gap by proposing a deep learning model that combines CNN with residual and attention mechanisms, specifically adapted for metaverse financial environments. The goal is to improve risk classification and support financial institutions in securely leveraging metaverse technologies.

## Materials and methods

In this section, we explain the main techniques and components used to build our model. We start with the 1D Convolutional Neural Network (1D-CNN), which helps extract important features from the input data. Then, we use a Residual Network (ResNet) to improve learning and avoid common issues like vanishing gradients. Finally, we add an Attention Mechanism to help the model focus on the most relevant parts of the data, making the overall system more accurate and efficient.

### 1D convolutional neural networks

A variant of 2D CNNs is known as 1D CNNs. The 1D CNN is a specific type of deep neural network. It has been developed and is particularly effective for analyzing textual data, time series, and other sequential data types^13^. In the dynamic environment of metaverse financial transactions, data is characteristically multivariate time series. It is generated from various user activities, transaction types, and virtual asset movements. These interconnected data streams capture complex behavioral patterns over time. For effective anomaly detection and risk classification in such dynamic environments, it is crucial to identify both non-linear and non-periodic characteristics within these short-term and long-term data flows. While Recurrent Neural Network (RNN) based models are widely recognized for time series analysis, their primary limitation in this domain is their tendency to focus solely on temporal features, potentially overlooking crucial spatial relationships within multivariate transaction data. One-dimensional Convolutional Neural Networks (1D-CNNs) are highly effective for multivariate time series data. Their kernels move along the time dimension, enabling the extraction of spatial features patterns across different variables at a given time point or local patterns within a sequence. The fundamental architecture of a 1D-CNN is demonstrated in^14^.

1 $$x_{j}^{l} = f\left( {\sum\nolimits_{{i \in M_{j} }} {x_{i}^{{l - 1}} \times k_{{ij}}^{l} + xb_{j}^{l} } } \right)$$

Where *x*_*i*_ is the input, $\:k$ is the kernel weight, *b* denotes the bias, $f(\cdot)$ denotes the activation function. *x*_*i*_ is the output of the *j*_*th*_ kernel in the *l*_th_ convolutional layer.

### Residual network

Residual networks^9^ are commonly referred to as ResNets. It is a deep neural network architecture intended to focus on the problem of vanishing gradients in very deep neural networks. This issue occurs when gradients become too small during training and make it difficult for earlier layers to update their weights effectively. ResNets presents residual connections that allow gradients to flow more easily during training, thereby preserving and enhancing gradient information throughout the layers. These residual connections allow more efficient training of very deep networks by enabling the direct flow of gradients. As a result, ResNets has significantly enhanced the training of very deep neural networks, leading to higher performance in various AI applications^23,24^. The core of ResNets is the residual block, which can be mathematically represented as shown in Eq. 2.

2 $$\:y=F(k,\{{w}_{i}\left\}\right)+k$$

Where *k* is the input of the residual block. (k,{ $\:{w}_{i}$}) represents the residual function typically composed of two convolutional layers. { $\:{w}_{i}$} are the weights of the layers in the residual function, and is the output of the residual block.

### Deep neural networks with attention mechanisms

Deep neural networks with attention mechanisms^13^ can concentrate on specific input features while disregarding others. A little research has discovered the utilization of attention mechanisms in deep neural networks for transaction data. These mechanisms are inspired by human visual attention^25^. By dynamically adjusting the importance of different input features, attention mechanisms improve the model interpretability to enhance decisions based on the most relevant features. Consequently, integrating attention mechanisms with 1D-CNNs not only enhances classification, accuracy and efficiency^26^.

## Dataset overview and characteristics

**Fig. 1:**
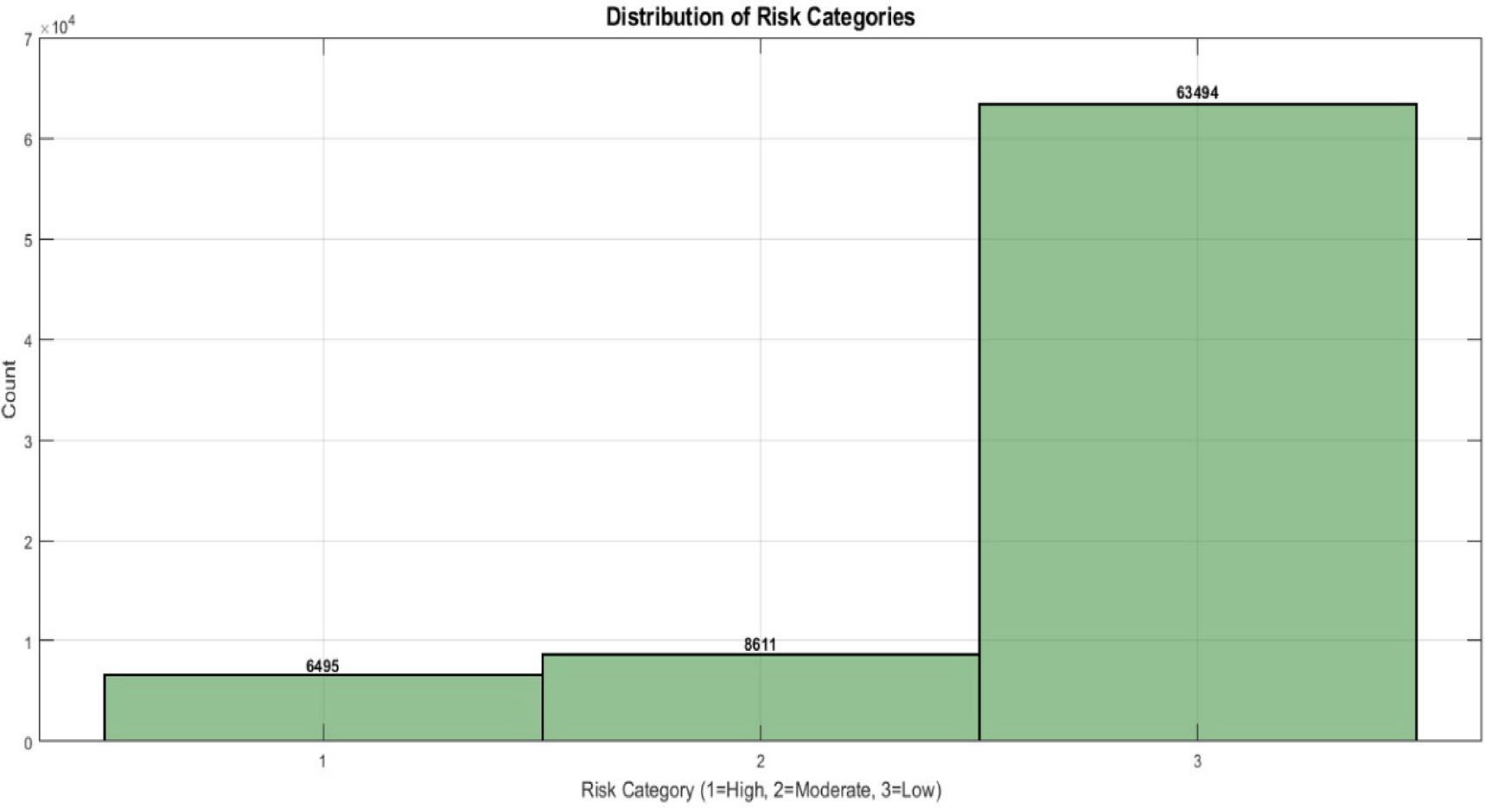
The class distribution of metaverse financial dataset.

The data utilized in this research is obtained from the metaverse transaction dataset from Kaggle^27^. This dataset presents blockchain financial transactions specifically considered for developing and testing anomaly detection within the open metaverse. With a focus on practical financial applications, this dataset captures a wide range of transaction types, user behaviors, and risk profiles across a global network. The original dataset is saved as a.*CSV* file. It contains 78,600 records, each detailing a transaction within the metaverse. Table 1 outlines 14 features of these transactions. The Anomaly attribute is divided into three categories labeled as “low_risk”, “moderate_risk”, and “high_risk”, as illustrated in Fig. 1.

**Table 1:** Dataset Characteristics.

ID	Feature name	Description
1	Timestamp	Date and time of the transaction.
2	Hour of day	Hour part of the transaction timestamp (0–23)
3	Sending address	Blockchain address of the sender.
4	Receiving address	Blockchain address of the receiver.
5	Amount	Transaction amount in a simulated currency.
6	Transaction type	Categorization of the transaction (e.g., transfer, sale, purchase, scam, phishing).
7	Location region	Simulated geographical region of the transaction.
8	IP prefix	Simulated IP address prefix for the transaction.
9	Login frequency	Frequency of login sessions by the user, varying by age group.
10	Session duration	Duration of activity sessions in minutes.
11	Purchase pattern	Behavioral pattern of purchases (e.g., focused, random, high-value).
12	Age group	Categorization of users into new, established, and veteran based on their activity history.
13	Risk score	Calculated risk score based on transaction characteristics and user behavior.
14	Anomaly	Risk level assessment (e.g., “low_risk”, “moderate_risk”, “high_risk”).

**Fig. 2:**
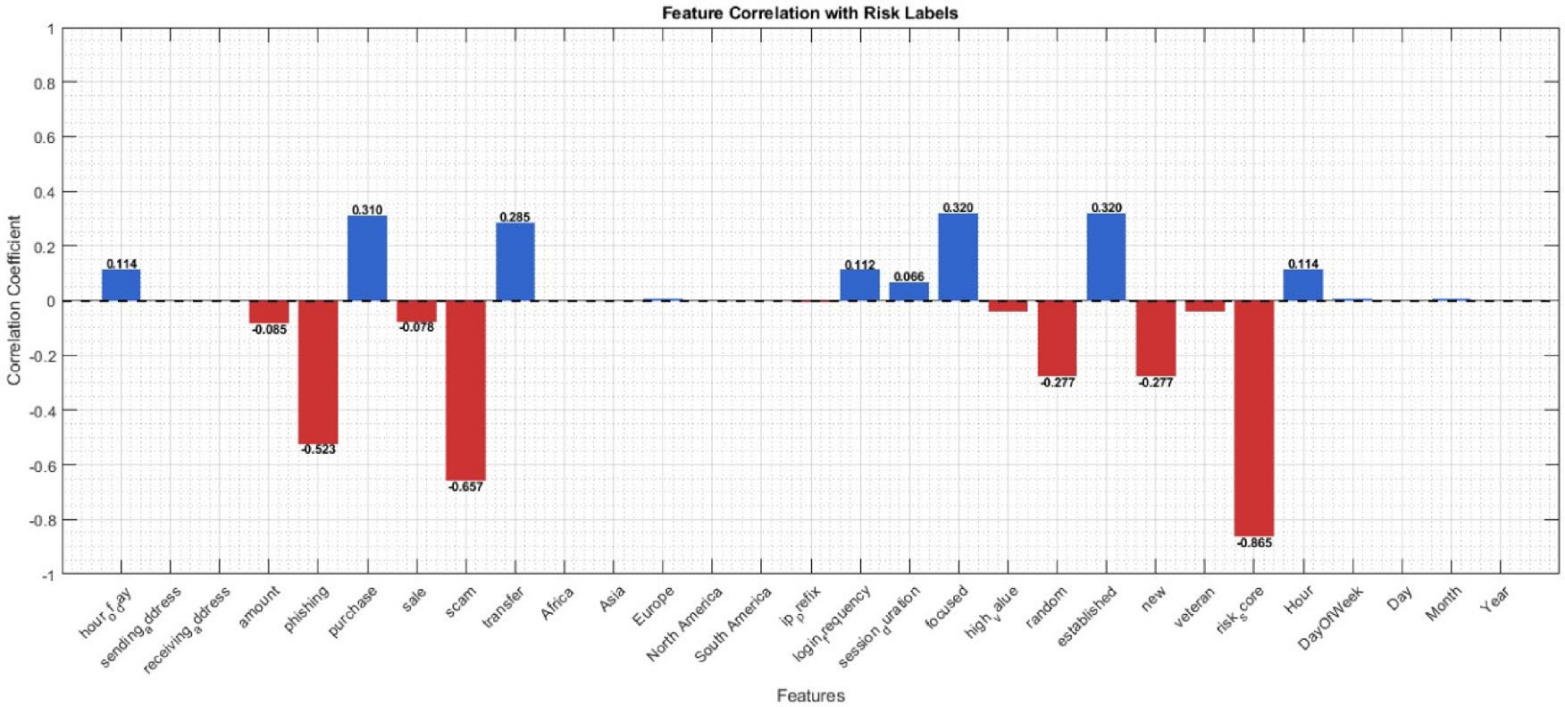
Correlation Coefficient with label of metaverse financial dataset.

The correlation coefficients between dataset features and the target labels in metaverse financial transactions are displayed in Fig. 2. The bars within the chart represent each feature’s correlation coefficient. Features showing positive correlation coefficients (blue bars above the x-axis) correlate with the target labels; that is, both tend to increase together. Conversely, features with negative correlation coefficients (red bars below the x-axis) have a relationship where feature value increments lead to decrements in target label values.

**Fig. 3:**
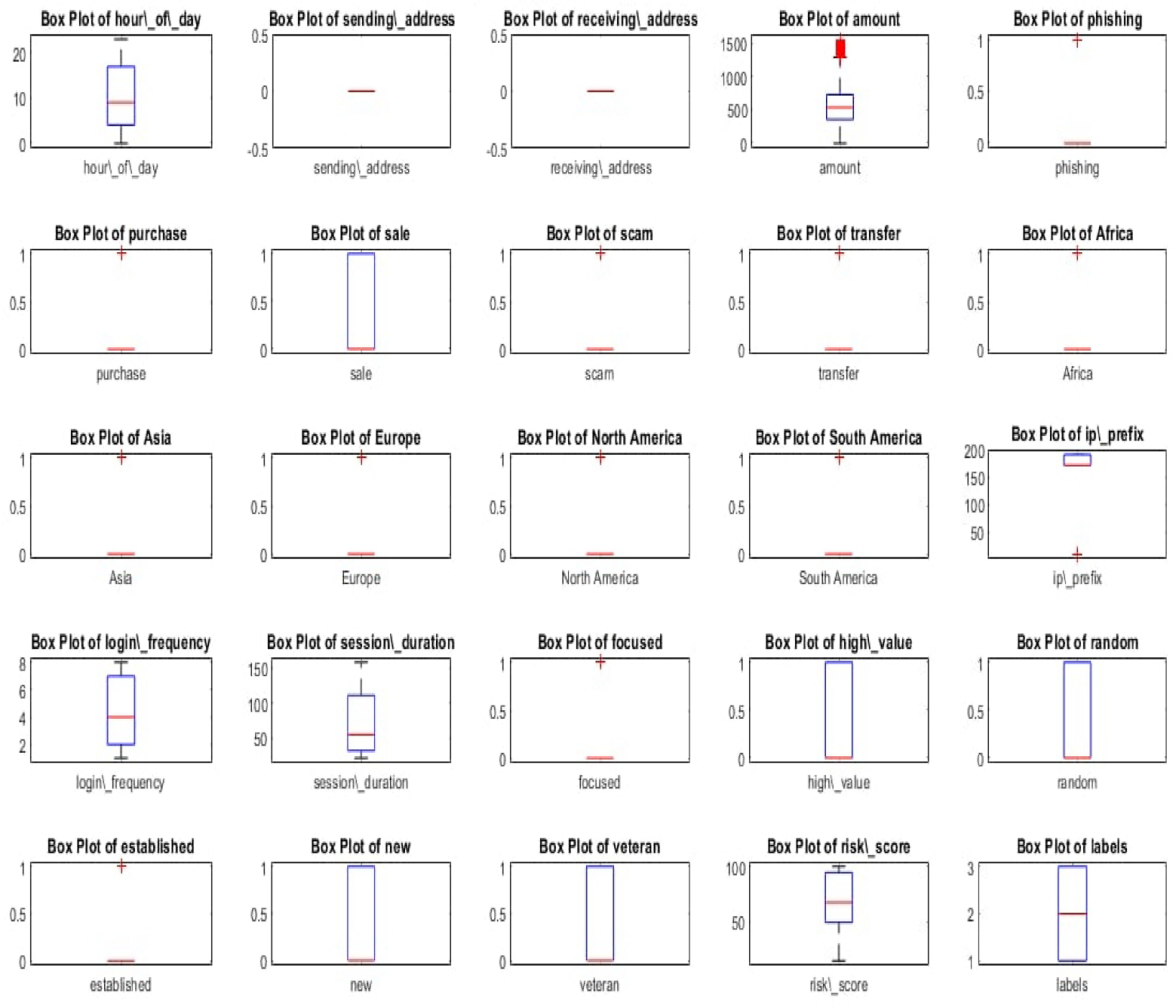
Boxplots for all features of metaverse financial dataset.

This visualization presented in Fig. 3 helps shed light on how data points are spread throughout different ranges as well as revealing relevant outliers. Overall, box plots provide details about central tendency, dispersion concerning range, interquartile range, alongside outlining potential outliers of every feature’s distribution. This can be vital during analysis of records prior to initial exploration and guided preprocessing procedures for enhanced clarity including but not limited to change treatments for outlying values (removal/replacement), adjusting values on a defined scale (feature scaling), treating categorical variables by assigning numerical representations post encoding processes amongst others.

## Proposed financial transactions classification model in metaverse

**Fig. 4:**
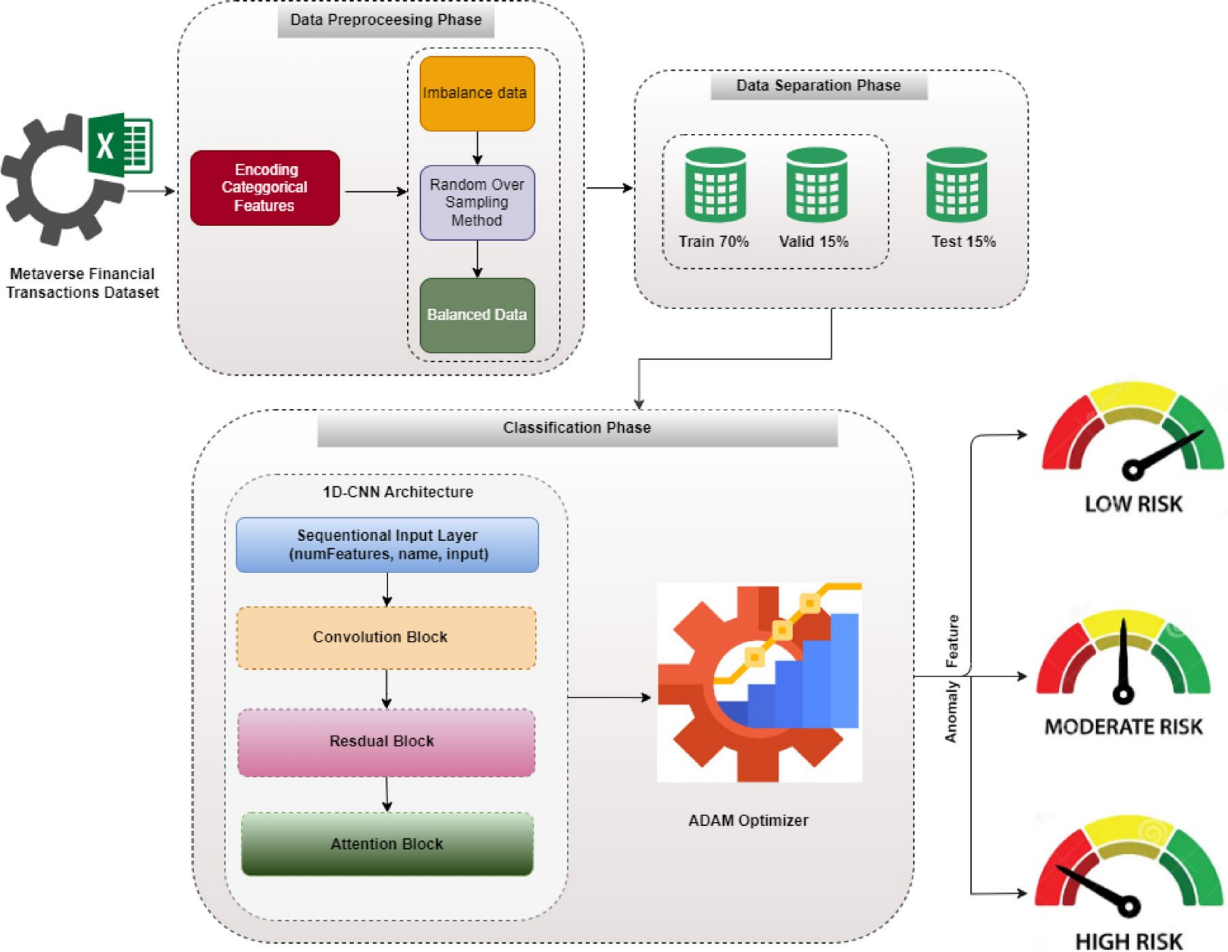
The proposed financial transactions classification model in metaverse.

This paper proposes a novel deep learning model utilizing a 1D-CNN with ResNet and attention mechanism. The objective of the proposed model is to classify the risk level of metaverse transactions in real-time based on historical behavioral patterns. The proposed model includes three main phases such as preprocessing, data separation, and classification. In the preprocessing phase, the dataset involves several preparation steps. The preparation steps include oversampling and categorical encoding, to ensure it is ready for analysis. The data separation phase involves splitting the dataset into a training set and testing set. Finally, the 1D-CNN architecture is applied to efficiently classify the risk levels of metaverse transactions in classification phase. The structure of the proposed model is illustrated in Fig. 4.

### Data preprocessing phase

In preprocessing data, two data preprocessing tasks are executed to enhance the quality of the dataset for analysis and modeling. One essential step involves feature encoding, which transforms categorical variables into numerical representations using One-Hot Encoding^28^. Additionally, data oversampling techniques can be applied to address class imbalance issues in classification tasks^29,30^. By increasing the representation of minority classes through oversampling, the model can learn more effectively from the entire dataset, leading to more balanced and reliable predictions.

#### Encoding categorical features

The paper investigates the impact of feature encoding method on the dataset used. The dataset features were divided into two categories are categorical features and numerical feature. The features such as “Transaction Type”, “Location Region”, “Purchase Pattern, Age Group, Anomaly were converted to numerical data type. For example, the “Age Group” of the sender was originally categorical and needed to be digitally encoded since computers can only process numeric inputs. This research implemented One-Hot Encoding, a common method for converting categorical features into numerical values without sequential information^28^. This method converts each categorical feature into a One-Hot binary vector, with 1 representing the relevant category and 0 representing all other categories. The encoded variables were split into separate columns. For example, “Age Group” feature contains two categories, each corresponding to a one-hot vector are “established” = {1} and “veteran” = {0}.

**Fig. 5:**
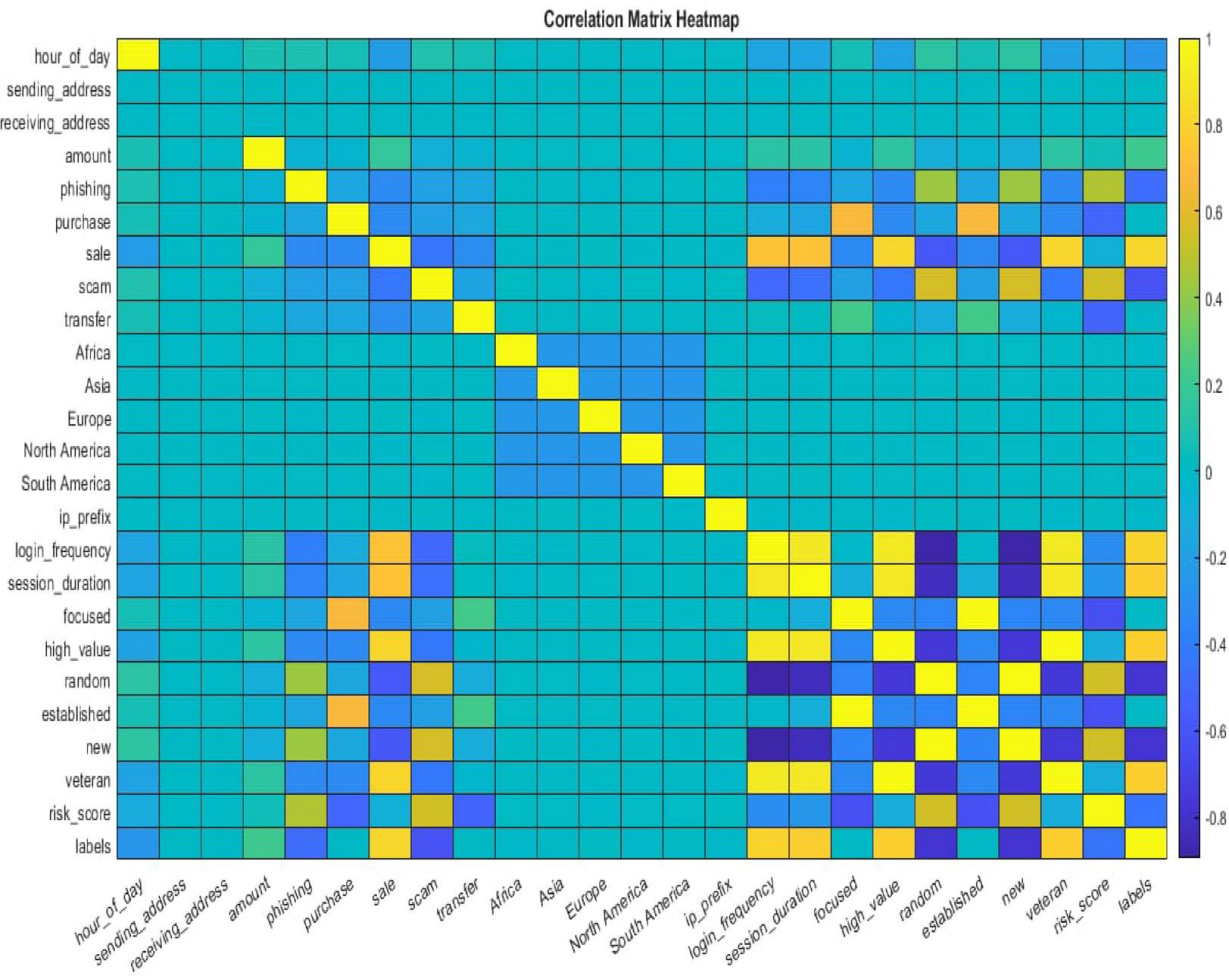
Features heatmap correlation of metaverse financial transactions dataset.

Similarly, the dataset contains some sequential features among categories, such as “location_region” which include five categories such as “Africa”, “Asia”, “Europe”, “North America”, and “South America”. For this type of feature, One-hot Encoding is not suitable. Instead, Sequential Encoding was used, mapping sequential features to different natural numbers. For example, “location_region” categories were mapped as follows: “Africa = {1}, Asia = {2}, Europe = {3}, North America = {4}, and South America = {5}”. Besides categorical features, the dataset al.so included numerical features, such as “Hour of Day,” representing the hour part of the transaction timestamp. Directly inputting these numerical features into the model can cause issues due to differences in the magnitudes of different attributes^31^. Figure 5 shows a correlation heatmap illustrating the significance of different features. In the correlation matrix, each row and column represent a continuous variable, and the values indicate the correlation coefficient between the variables represented by the corresponding row and column. The observations indicate a significant correlation between most attributes.

#### Data oversampling

The dataset was resampled to balance the class distribution. The target “Anomaly” feature is divided into three risk levels assessment are “low_risk”, “moderate_risk”, and “high_risk”. The “low_risk” with 63,494 records, “moderate_risk” with 8611 records, “high_risk” with 6495 records. The significant difference in class distribution set challenges for achieving high-accuracy models in classification tasks. To address this imbalance in the dataset, Random Over-Sampling (ROS) method was employed. This method generates new instances by randomly replicating original instances from the minority classes (moderate_risk and high_risk) to match the total instances from the majority class (low_risk)^29,30^. This resulted in a balanced dataset with 63,494 samples for each class. After oversampling, the data was mixed and then split into training (70%), validation (15%) and test (15%) datasets.

### Proposed one-dimensional (1D-CNN) architecture

**Fig. 6:**
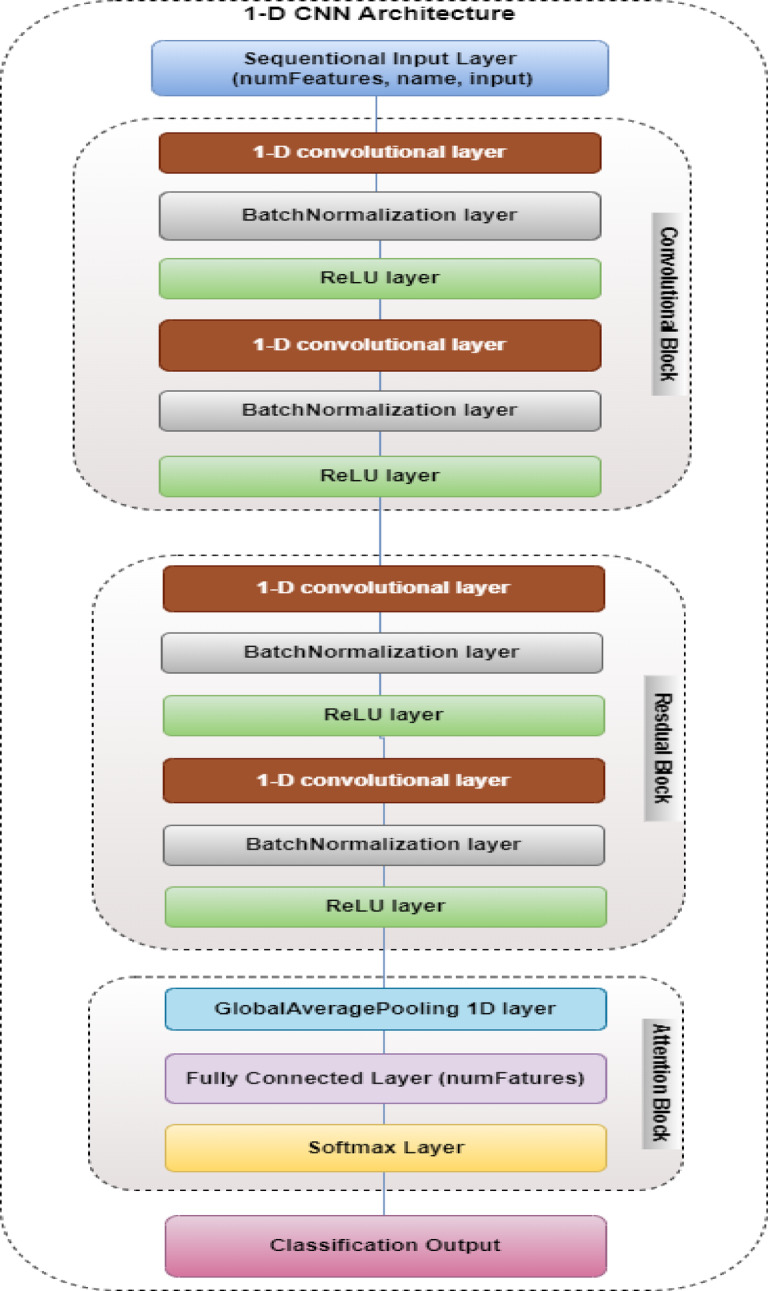
Proposed 1D-CNN architecture model.

A novel 1D-CNN architecture was proposed. It integrates residual connections and an attention mechanism. The architecture of the proposed 1D-CNN is designed to capture the sequential nature of transaction data effectively as shown in Fig. 6. This architecture involves a sequential input layer followed by three main blocks are convolutional, residual, and attention blocks. The sequential input layer, which takes the input features and forwards them to the next three blocks sequentially. Each block has a number of layers to finally generate classification output.

As illustrated in Fig. 6, convolutional layer blocks are fundamental components of the 1D-CNN. The 1D-CNN serves as the foundation of this architecture. It provides excellent capability in processing sequential financial transaction data. Through the systematic application of sliding convolutional filters, these networks extract hierarchical features that range from simple local patterns to complex abstract representations. This multi-level feature extraction enables the identification of subtle anomalies that may indicate fraudulent activity, while maintaining computational efficiency essential for real-time processing. The specialized adaptation of 1D-CNNs to temporal data sequences ensures optimal performance in capturing the intrinsic patterns within transaction streams.

#### Convolutional block

The convolutional block contains three layers. Firstly, there is a 1D Convolutional Layer, which applies convolutional filters to extract features from the input data^32^. Secondly, batch normalization layer, which normalizes the output of the convolutional layer to enhance training stability and convergence as shown in Eqs. 3 and 4.

3 $$\:{\mu\:}_{B}=\frac{1}{m}\sum\:_{i=1}^{m}{x}_{i}$$

4 $$\:{\sigma\:}_{B}^{2}=\frac{1}{m}\sum\:_{i=1}^{m}{{(x}_{i}-{\mu\:}_{B})}^{2}$$

where $\:B\:$denotes the mini batch of size m.

Finaly, there is a Rectifying Linear Activation Units (ReLU) layer, which applies to the ReLU activation function^33^. This layer breaks the linear nature of the network by setting negative values within the network to zero. The ReLU function is defined mathematically as shown in Eq. 5 below.

5 $$\:\left(x\right)=\left\{\begin{array}{c}0\:\:\:for\:x<0\\\:x\:\:\:\:for\:x\ge\:0\end{array}\right.$$

#### Residual block

The residual block consists of two 1D convolutional layers, each followed by batch normalization and ReLU activation. Each convolutional layer uses 64 filters with a kernel size of 3. The batch normalization layers normalize across 64 channels, helping to stabilize training. Residual connections allow for efficient training of deeper networks by addressing the vanishing gradient problem, providing a direct path for gradients to flow through the network^9^. This structure enables the network to learn residual functions relative to the layer inputs, potentially enhancing performance in deep architectures.

#### Attention block

The attention block includes a Global Average Pooling 1D layer. This layer consolidates features into sub-maps with prominent characteristics, enhancing information quality^25^. Following global average pooling, an attention mechanism is employed using fully connected layers with a SoftMax activation to generate attention weights. The final classification is performed by a fully-connected output layer and a SoftMax activation function for multi-class classification^34^. This layer takes the output of the global average pooling layer and combines all the features into a single vector as shown in Eq. 6 below.

6 $$\:{k}_{pj}=\:\sum\:_{i=1}^{m}{x}_{i}^{l-1}\:{\mathbb{W}}_{ji}^{l}+\:{b}_{j}^{l}$$

where $\:{x}_{i}$ is an input features, $\:m$ is the total number of input features to neuron $\:i$ in the current layer. $\:{\mathbb{W}}_{ji}^{l}$ represent the weight of connection from neuron $\:i$ in layer $\:l-1$ to neuro $\:j$ in layer $\:l$, and $\:{b}_{j}^{l}$ is the bias of neuron $\:j$ in layer $\:l.$ The $\:{k}_{pj}$ represent weighted sum that passed via an activation function to generate final output of the neuron^1–3^.

For multi-class classification, a SoftMax layer is used as shown in Eq. (7). This layer applies the SoftMax activation function which convert values into probabilities that sum up to 1 for representing the probability of each class^35^. A self-attention mechanism, realized through a SoftMax layer, which assigns higher weights to the most relevant features for risk classification.

7 $$\:\boldsymbol{\sigma\:}{(\overrightarrow{\boldsymbol{z})}}_{\boldsymbol{i}}=\frac{{\mathrm{e}}^{{\mathrm{z}}_{\mathrm{i}}}}{\sum\:_{\mathrm{j}=1}^{\mathrm{k}}{\mathrm{e}}^{{\mathrm{z}}_{\mathrm{j}}}}$$

Where $\:\overrightarrow{\mathrm{z}}$ represents the input vector, $\:k$ is the number of classes, $\:{\mathrm{e}}^{{\mathrm{z}}_{\mathrm{i}}}$ is the exponential function for input vector, $\:{\mathrm{e}}^{{\mathrm{z}}_{\mathrm{j}}}$ is the exponential function of output vector.

The classification output is the final output of the proposed classification 1D-CNN architecture. It provides the predicted class probabilities for the input. This architecture combines convolutional layers for feature extraction with global average pooling and fully connected layers for classification. During the training, the classification layer uses the output from the SoftMax function to allocate input to one of the *K* distinct classes. This allocation is based on the cross-entropy function, employing a 1-of-M coding scheme^36^. The binary cross entropy loss function is generally used. It is defined as:

8 $$Z_{{binary}} = - \frac{1}{M}\sum {_{{j = 1}}^{M} } \left( {y_{j} {\mathrm{log}}\hat{y}_{j} + (1 - y_{j} ){\mathrm{log}}(1 - \hat{y}_{j} )} \right)$$

where $\:M$ is the class number, $\:{y}_{j}$ ∈ {0,1} is the target value, $\hat{y}_{j}$ ∈ [0,1] is the predicted score.

The proposed model was trained using Adam optimizer, an optimization algorithm that update the weights of the network iterative during the training data^37^. Table 2 outlines the various parameters employed during the model training. The learning rate was set 0.001 represents the percentage at which weights are modified. It was reduced by a factor of 0.0005 over time. Training was performed over 100 epochs, with categorical cross-entropy utilized as the loss function. The batch size determines the number of samples used in one epoch to train the neural network, was set to 16.

**Table 2:** Parameters values utilized by optimizer.

Parameter	Value
Optimizer	Adam
Learning rate	0.0005
Metric	Accuracy
Batch size	16
Epochs	100

### Performance evaluation

The effectiveness of the proposed model was assessed utilizing evaluation metrics such as Accuracy, Error_Rate, Sensitivity, Precision, False Positive Rate, and F1_Score^38,39^.

9 $$\:Accuracy=\:\frac{TP+TN}{TP+\mathrm{T}\mathrm{N}+FN+FP}$$

10 $$\:Error\_Rate=\:\frac{FP+FN}{N}$$

11 $$\:Sensitivity=\:\frac{TP}{TP+FN}$$

12 $$\:Precision=\:\frac{TP}{TP+FP}$$

13 $$\:False\:Positive\:Rate=\:\frac{FP}{TN+FP}$$

14 $$\:F1\_Score=\:\frac{2TP}{2TP+FP+FN}$$

where $\:TP$ is True Positive, $\:FP$ is False Positive, $\:FN$ is False Negative, $\:TN$ is True Negative, and $\:N$ is total number of cases.

## Experimental results and evaluations

The results and evaluation metrics of the experiments of the proposed model are discussed in this section. The experiments utilized a GPU optimized for MATLAB R2022b software. Every experimental process is conducted on a computer through core i7 processor and 16 GB of RAM, with GPU managing all experimental tasks.

### Training progress and accuracy

**Fig. 7:**
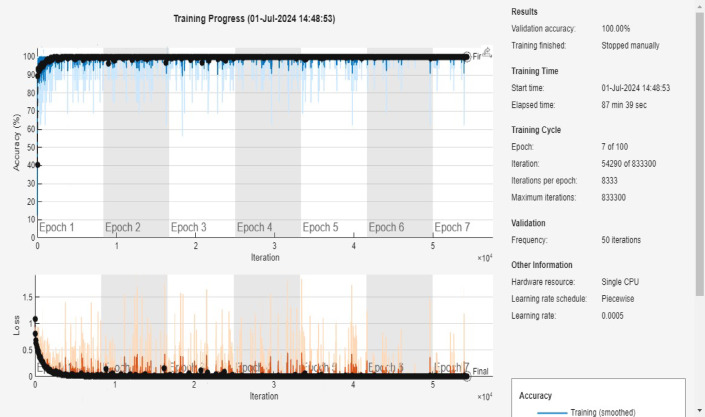
Training progress accuracy and loss curves for 3 risk classes.

As shown in Fig. 7, the proposed model was trained with a mini-batch size of 16 samples per iteration. Such a small batch naturally requires many iterations to complete an epoch on a large dataset. Furthermore, to address class imbalance the random oversampling was applied. It effectively increases the training data size via repetition of minority-class samples. After applying random oversampling, the dataset expanded to 190,482 samples in total (each of the risk classes was equalized to 63,494 samples each). This balancing dramatically increased the number of training examples compared to the original dataset. Following oversampling, the data was shuffled and split into training (70%), validation (15%), and test (15%) sets. The training set contained approximately 133,337 samples (70% of 190,482). The iterations per epoch with ~ 133,337 training samples and batch size 16, each epoch consisted of roughly 8,333 iterations (133,337 ÷ 16 ≈ 8,333). In other words, the model had to iterate over ~ 8.3k mini batches to cover the training set once. This value matches the “Iterations per epoch: 8333” shown in the training log. Over multiple epochs, the iteration count accumulates accordingly. The training was originally configured for up to 100 epochs, which corresponds to a theoretical maximum of ~ 833,300 iterations (8,333 iterations/epoch × 100). The reference to “833,000” iterations in the manuscript was a projected upper limit in the training configuration. In practice, however, we manually stopped training after 7 epochs, once the model had clearly converged. By epoch 7, roughly 58,331 iterations (7 × 8,333) had been executed only about 7% of the configured maximum. Thus, the high iteration number reflects the potential iterations for 100 epochs, not the actual iterations run for the 7-epoch training.

### Confusion matrix and classification performance

**Fig. 8:**
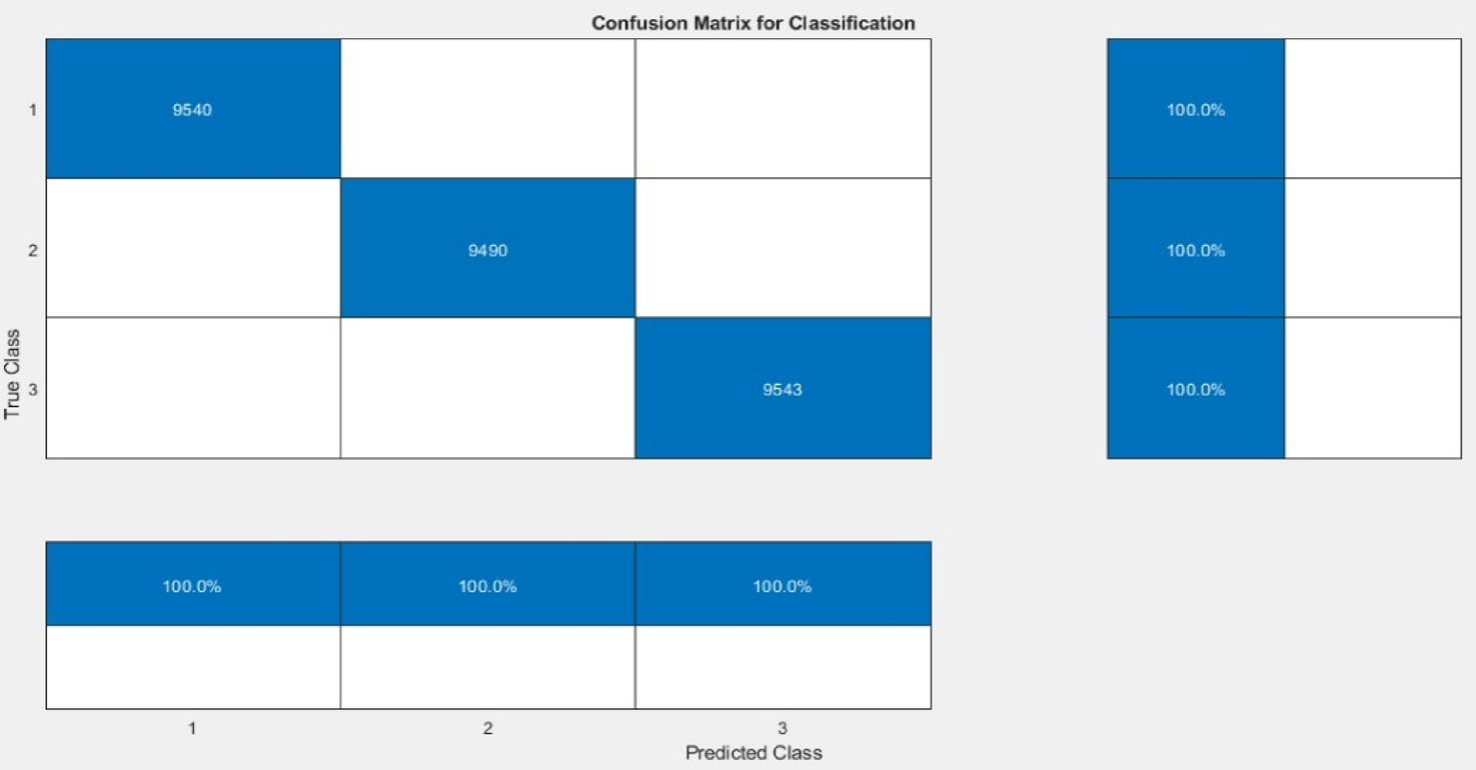
The confusion matrix of three classes in testing Accuracy Results.

As shown from Fig. 8, the confusion matrix for the test dataset demonstrated perfect accuracy across all three risk classes (“low_risk”, “moderate_risk”, and “high_risk”). The 3 × 3 matrix shows perfect accuracy, with 9940, 9490, and 9543 correct predictions for classes 1, 2, and 3 respectively. The proposed model achieved 100% precision and recall for each class, indicating a highly effective classification model with a strong feature set.

### The 5-fold cross-validation performance and statistical analysis

The implementation of stratified 5-fold cross-validation using MATLAB cvpartition function with the ‘Stratify’ option is to ensure related class representation across all folds. The complete results are presented below as shown in Table 3. The cross-validation results show significant consistency across all five folds, with standard deviations of only 0.0001 (0.01%) for accuracy, precision, recall, and F1-score. This minimal variance indicates stable model behavior rather than overfitting to any particular data split.

**Table 3:** Stratified 5-fold cross-validation performance metrics.

Fold	Accuracy	Precision	Recall	F1-Score	AUC-ROC
1	1.0000	1.0000	1.0000	1.0000	1.0000
2	0.9998	0.9998	0.9998	0.9998	1.0000
3	1.0000	1.0000	1.0000	1.0000	1.0000
4	1.0000	1.0000	1.0000	1.0000	1.0000
5	1.0000	1.0000	1.0000	1.0000	1.0000
Mean ± Std	1.0000 ± 0.0001	1.0000 ± 0.0001	1.0000 ± 0.0001	1.0000 ± 0.0001	

To determine the appropriate statistical tests for our cross-validation results, we first assessed the normality of performance metric distributions using the Shapiro-Wilk test^40^. All metrics exhibited p-values greater than 0.05. This indicates the distributions do not significantly deviate from normality. However, given the small sample size (*n* = 5 folds) and the careful nature of our analysis, we additionally employed non-parametric tests as shown in Table 4.

**Table 4:** Shapiro-Wilk normality test results.

Metric	W-Statistic	*p*-value	Distribution
Accuracy	0.5307	0.3032	Normal
Precision	0.5307	0.3032	Normal
Recall	0.5307	0.3032	Normal
F1-Score	0.5307	0.3032	Normal
AUC-ROC	0.5307	0.3032	Normal
Sensitivity	0.5307	0.3032	Normal
Specificity	0.5307	0.3032	Normal

Then, Wilcoxon signed-rank tests comparing fold performance against a baseline threshold of 0.90^41^. The p-value of 0.0625 considers but does not reach statistical significance at α = 0.05. This is expected given that with only 5 folds, the minimum achievable p-value for the Wilcoxon signed-rank test is 0.0625 when all observations exceed the comparison value. Significantly, all performance metrics across all folds exceeded the 0.90 baseline threshold, confirming consistently excellent performance as shown in Table 5.

**Table 5:** Wilcoxon signed-rank test results.

Metric	W-Statistic	*p*-value	Significance	Above 0.90?
Accuracy	15.0	0.0625	Not Sig.	Yes
Precision	15.0	0.0625	Not Sig.	Yes
Recall	15.0	0.0625	Not Sig.	Yes
F1-Score	15.0	0.0625	Not Sig.	Yes
AUC-ROC	15.0	0.0625	Not Sig.	Yes
Sensitivity	15.0	0.0625	Not Sig.	Yes
Specificity	15.0	0.0625	Not Sig.	Yes

### t-SNE visualization

**Fig. 9:**
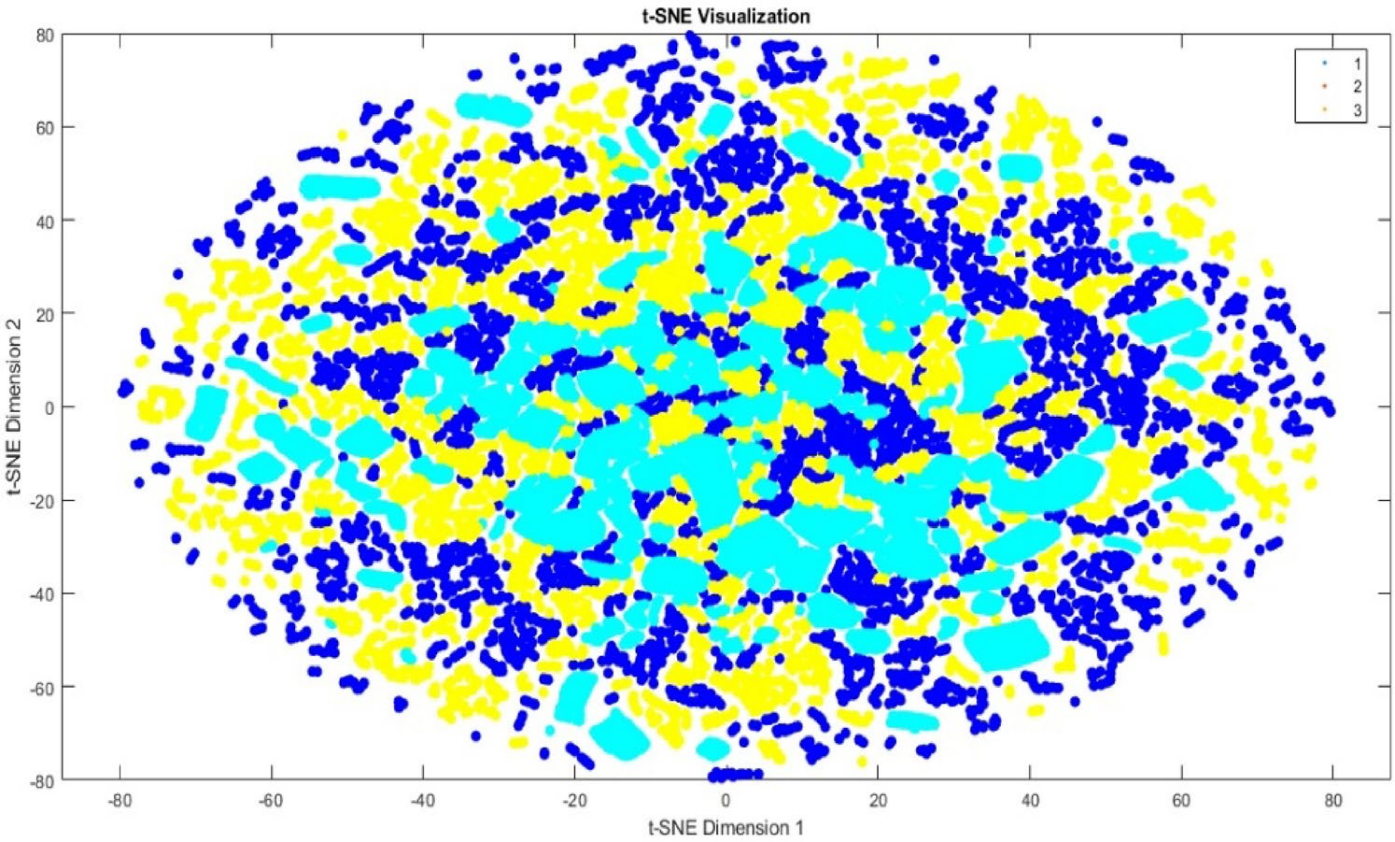
The t-SNE illustration of features for 3-class before classification.

**Fig. 10:**
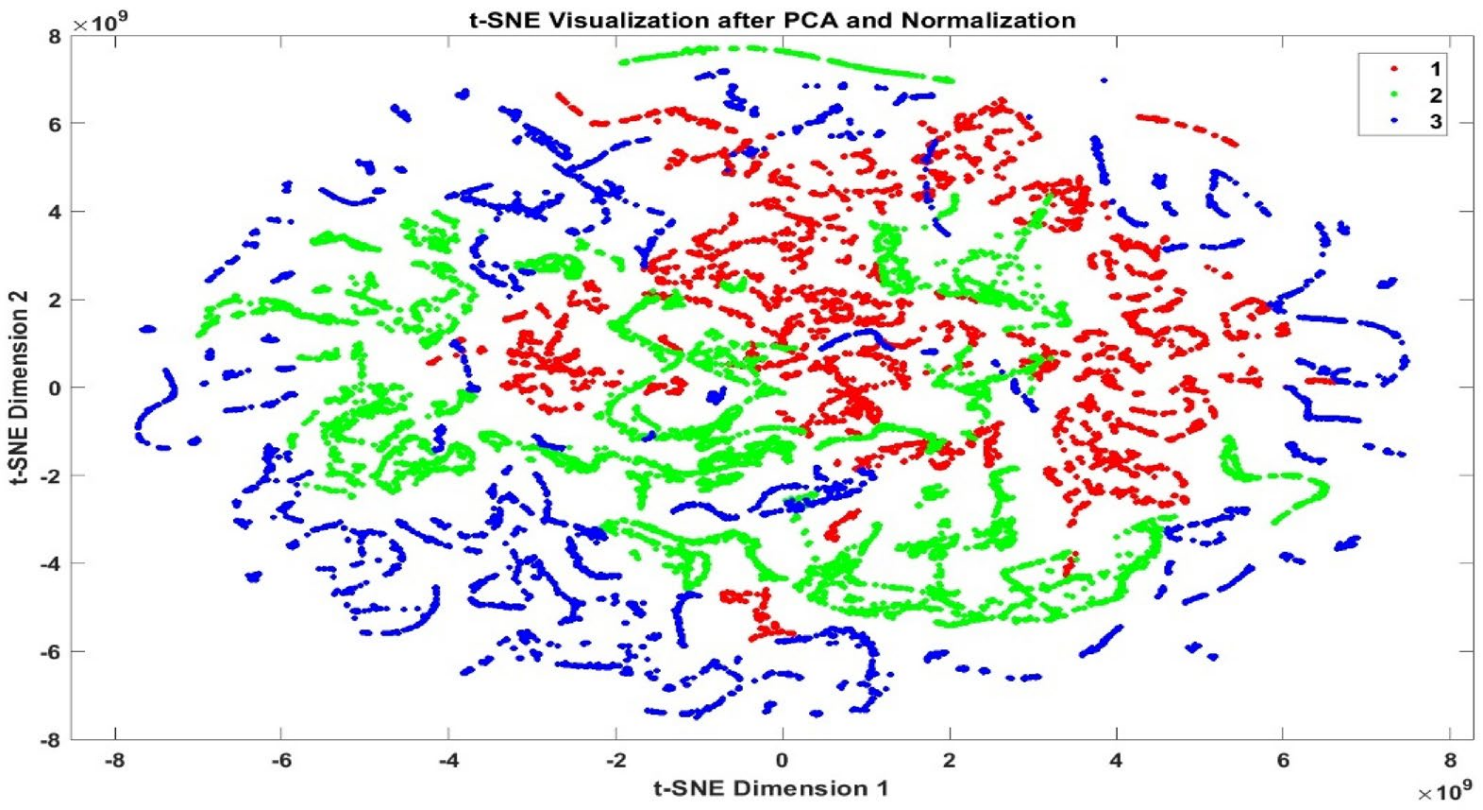
The t-SNE illustration of features for 3-class after classification.

To further evaluate the performance of the classification model, t-Distributed Stochastic Neighbor Embedding (t-SNE) was employed to visualize high-dimensional feature representations in a two-dimensional space^37^. Figures 9 and 10 illustrate the t-SNE charts of the features before and after classification, respectively, corresponding to the three risk categories: *low_risk*, *moderate_risk*, and *high_risk*. Figure 9 shows the initial distribution of the risk classes in the feature space prior to classification, where the class boundaries are less distinct. In contrast, Fig. 10 presents the post-classification t-SNE visualization, revealing a clearer separation among the risk levels. This indicates the proposed model effectiveness in learning discriminative features and accurately distinguishing between different risk categories in metaverse transactions.

### Comprehensive feature importance analysis for metaverse transaction fraud detection

**Fig. 11:**
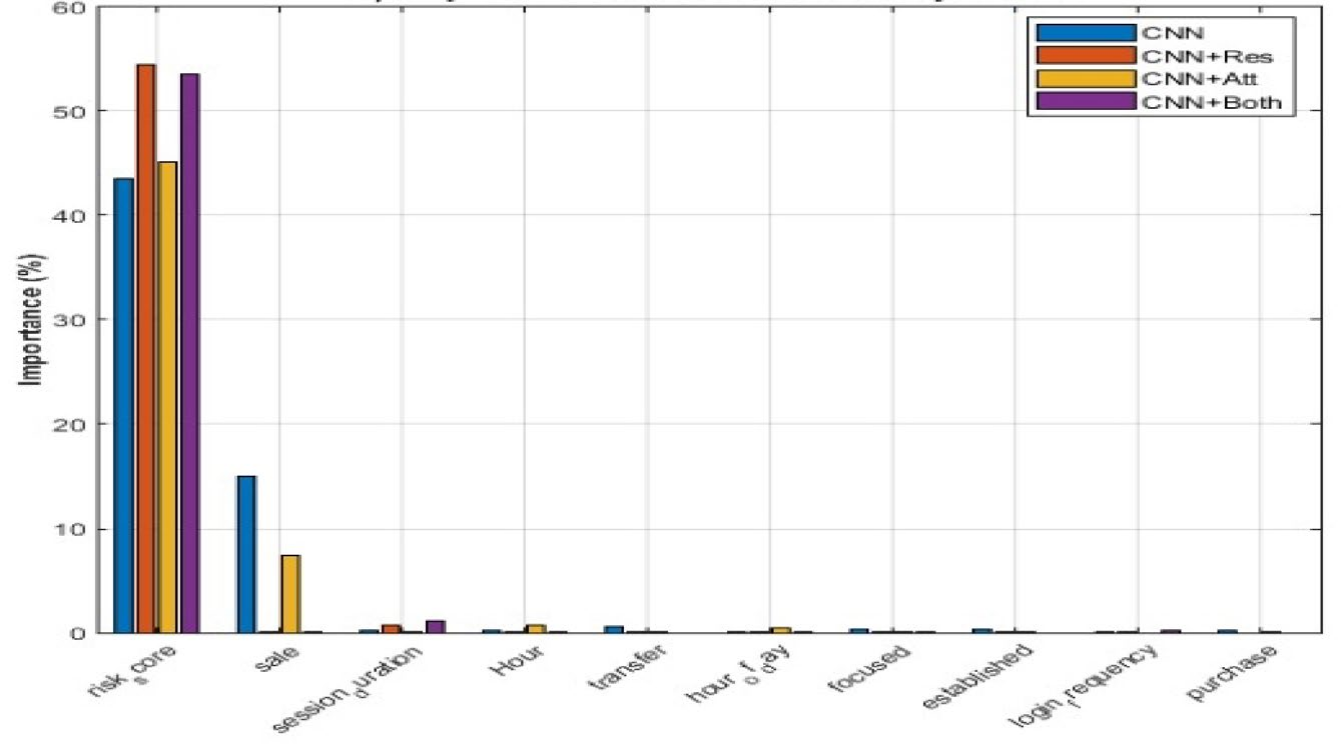
Comprehensive Feature Importance Analysis for each model.

The feature importance analysis conducted for detecting fraud in Metaverse transactions across different machine learning models as shown in Fig. 11. This figure provides a comparison of the importance of the top 10 features across four different models such as CNN, CNN + Res (Residuals), CNN + Att (Attention), and CNN + Both (Combination of Residuals and Attention). In this comparison, risk_core remains the dominant feature across all models. It is maintaining a substantial importance percentage of over 50%. The sale feature showed significant importance in the CNN + Res and CNN + Att models, while the session_duration feature displayed lower importance across all models, as indicated by its smaller contribution to the feature importance. This analysis demonstrates the critical features for detecting fraud in Metaverse transactions. It highlights the relevance of risk_core with various models contributing differently to the evaluation of features based on their unique architecture. In Table 6 shows the feature importance scores derived from three different model configurations.

**Table 6:** Feature importance analysis report.

Index	Feature name	Description	Importance_CNN_ plus_Residual	Importance_CNN_ plus_Attention	Importance_CNN_ plus_ Residual_plus_Attention
24	risk_score	Risk score	54.44	45.19	53.55
7	sale	Sale transaction indicator	0.03	7.38	0.03
17	session_duration	Session duration	0.69	0.07	1.09
27	Hour	Hour component	0.07	0.77	0.02
9	transfer	Transfer transaction indicator	0.00	0.09	0.00
1	hour_of_day	Hour of the day (0–23)	0.03	0.41	0.09
18	focused	Focused purchase pattern	0.03	0.02	0.00
21	established	Established user type	0.03	0.01	0.00
16	login_frequency	User login frequency	0.01	0.00	0.26
6	purchase	Purchase transaction indicator	0.00	0.04	0.00
4	amount	Transaction amount	0.04	0.00	0.01
20	random	Random purchase pattern	0.00	0.00	0.00
22	new	New user type	0.00	0.00	0.00
15	ip_prefix	IP address prefix	0.00	0.00	0.00
19	high_value	High value purchase pattern	0.00	0.00	0.00
23	veteran	Veteran user type	0.00	0.00	0.00
25	Seconds	Seconds component	0.00	0.00	0.00
26	Minutes	Minutes component	0.00	0.00	0.00
29	Day	Day of month	0.00	0.00	0.00
14	South_America	Transaction from South America	0.00	0.00	0.00
30	Month	Month number	0.00	0.00	0.00
2	sending_address	Sending address identifier	0.00	0.00	0.00
3	receiving_address	Receiving address identifier	0.00	0.00	0.00
5	phishing	Phishing transaction indicator	0.00	0.00	0.00
8	scam	Scam transaction indicator	0.00	0.00	0.00
10	Africa	Transaction from Africa	0.00	0.00	0.00
11	Asia	Transaction from Asia	0.00	0.00	0.00
12	Europe	Transaction from Europe	0.00	0.00	0.00
13	North_America	Transaction from North America	0.00	0.00	0.00
28	DayOfWeek	Day of week	0.00	0.00	0.00
31	Year	Year of transaction	0.00	0.00	0.00

### Ablation study: component-level analysis for the proposed 1D-CNN architecture

To better understand how each part of the proposed 1D-CNN architecture contributes to its overall performance, we carried out ablation Study of component-level analysis. This experiment focused on evaluating the individual and combined effects of three key components: the convolutional block, the residual block, and the attention mechanism. We tested four different model configurations:

- CNN only: A baseline model using only the convolutional block.
- CNN with Residual (CNN_R): Incorporates a residual block to enhance feature propagation and mitigate vanishing gradients.
- CNN with Attention (CNN_A): Combines the convolutional block with an attention mechanism to focus on the most informative features.
- CNN with Residual, and Attention (CNN_R_A): The full model integrating all three components for maximum performance.

**Fig. 12:**
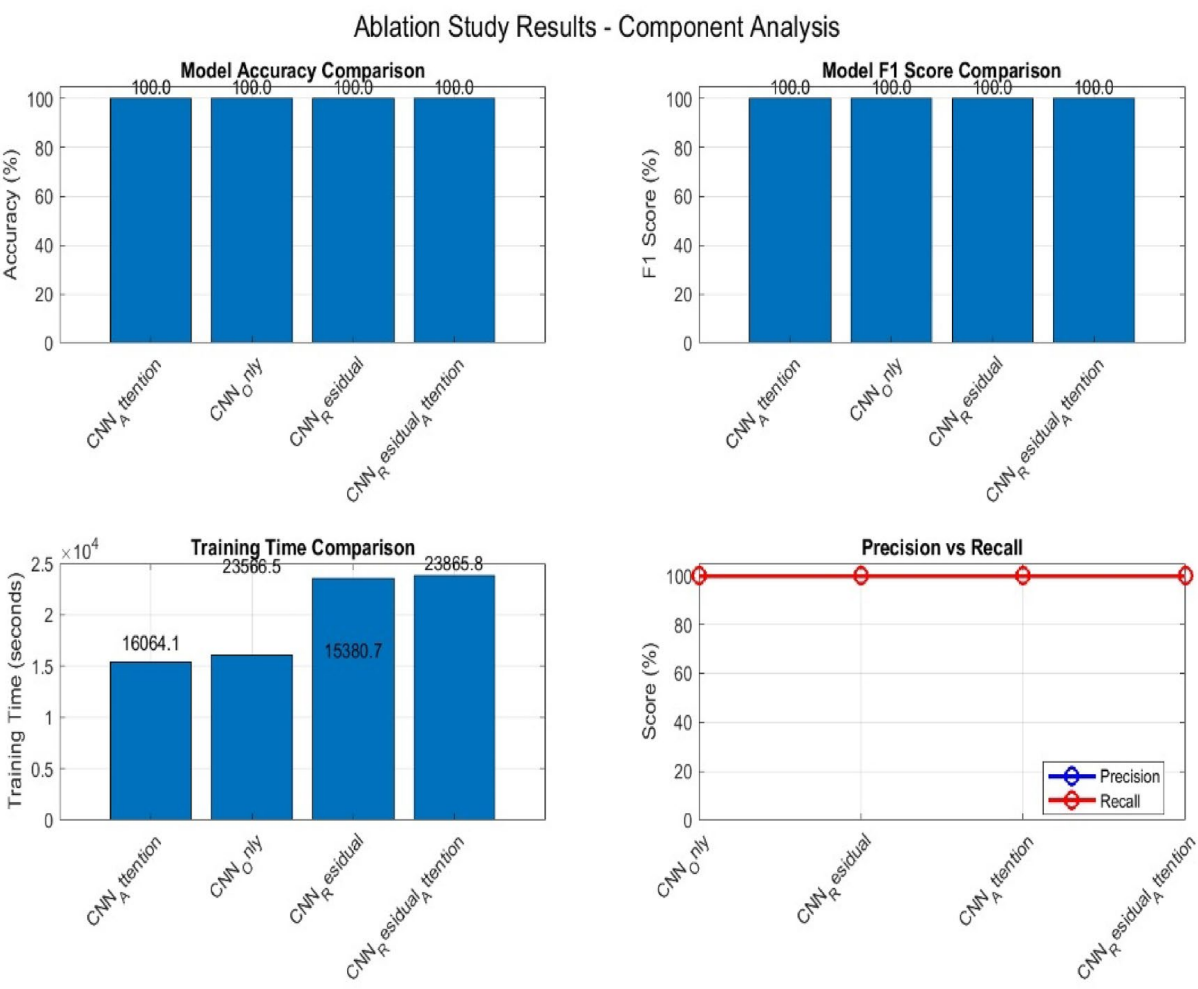
Ablation study results component analysis.

Each alternative was trained and evaluated under the same conditions using the oversampled metaverse dataset. The results performed over 190,482 samples, 30 features, and 3 classes of risk assessment including “low_risk, moderate_risk, and high_risk”. The Performance metrics used are accuracy and F1 Score. All model variants achieved accurate results 100% in both accuracy and F1 Score metrics, as shown in the top two plots of Fig. 12. This indicates that each component variant could successfully classify the three risk levels. However, these results highlight the importance of further generalization on external datasets to assess robustness. The training Time in CNN_A component stated the shortest training time (16,064.1 s), followed closely by CNN_R (15,380.7 s). The complete proposed model (CNN_R_A) had the highest training time (23,865.8 s). It reflects the computational overhead introduced by integrating both residual and attention mechanisms. Training time comparisons are presented in the bottom-left chart of Fig. 12. The evaluation of precision and recall is presented also in Fig. 12. All variants demonstrated 100% precision and 100% recall across three risk classes. This indicate no false positives, or false negatives were produced on the test dataset. These results are visualized in the bottom-right chart of Fig. 12.

While performance across all components remained stable. The differences in training times indicate that CNN_R offers the best trade-off between efficiency and performance. The complete proposed model (CNN_R_A) is optimal when interpretability (via attention) is prioritized and longer training time is acceptable. The results of ablation study are presented in Table 7.

**Table 7:** Results of ablation study.

Model	Accuracy	Precision	Recall	F1Score	Training time (s)
CNN_Only	100	100	100	100	16064.12072
CNN_Residual	100	100	100	100	23566.47466
CNN_Attention	100	100	100	100	15380.68266
CNN_Residual_Attention	100	100	100	100	23865.83755

The choice of the proposed 1D-CNN architecture with residual connections and an attention mechanism not just for accuracy, but for its robustness, generalizability, and interpretability. Although the ablation study showed that all components of the proposed 1D-CNN variants achieved the same excellent performance. There are strong reasons to prefer applying the complete 1D-CNN architecture on the metaverse financial transaction dataset. Residual connections help stabilize training by addressing vanishing gradients, making it easier to train deeper networks. This adds resilience, especially as data complexity increases. Attention mechanisms improve generalization by helping the model focus on the most relevant parts of the input, effectively filtering out noise. While the baseline CNN performed well on our current dataset, the overall proposed 1D-CNN architecture is better trained to handle more challenging or noisy scenarios.

In terms of interpretability, attention layers provide insight into which features affect the proposed model decisions. It is an important advantage in applications like financial anomaly detection, where understanding model behavior is critical. A plain CNN lacks this transparency. The combination of residual and attention modules also led to smoother and faster convergence during training, even if it didn’t improve final accuracy. This could be crucial in real-world applications where data is less clean, or training samples are limited. We admit that this enhanced model comes with a computational cost, increasing training time by about 48%. However, inference time remains low, and added reliability and explainability justify the trade-off. In simpler environments, a baseline CNN might sufficient, but for high-stakes applications, the complete hybrid proposed model offers a safer, and more future-proof choice. The proposed 1D-CNN architecture model breakdown showing the quantitative effect, qualitative benefits, and risk if omitted for each architectural element in the proposed CNN-based model architecture. This visualization supports the proposal reasoning of the full hybrid model (CNN + Res + Attn) used in metaverse risk classification tasks. Comparative breakdown of each contribution of model component, strategic advantage, and associated risk if excluded from the architecture as indicated in Table 8.

**Table 8:** Comparative breakdown of each 1D-CNN component architecture.

Component	Quantitative effect on current balanced dataset	Qualitative/strategic benefit	Risk if omitted
1 D CNN backbone	Learns local temporal/feature patterns; already achieves 100% accuracy and F1 on synthetic metaverse transactions.	Fast, highly parallelisable; well suited to 1 D sequential financial data.	—
Residual block	Adds + 47% training time but no loss of accuracy (100%).	Stabilises deeper networks (mitigates vanishing gradients). Allows rapid convergence (plateau by epoch 4). Acts as a “safety net” for future datasets that may require deeper receptive fields.	Without the skip connection, deeper variants can under train or overfit when data become noisy or class imbalanced in production.
Self-Attention layer	Adds < 5% training time; accuracy again unchanged at 100%.	Dynamically weights behavioural + contextual features (e.g., unusual avatar behaviour, rare virtual asset types). Provides built in interpretability—attention weights	Model becomes a “black box”; harder to justify automated blocking decisions to compliance teams. Missed long range cross feature interactions when patterns grow subtler.
Hybrid (CNN + Res + Attn)	+49% training time vs. CNN only; maintains perfect scores.	CNN captures fine grained sequential cues.– Residual block lets us stack filters confidently without degradation.– Attention surfaces global, cross feature anomalies and explains them.	Risk of deploying a “brittle” minimalist model that breaks when the live class distribution shifts, or when regulators demand feature level explanations.

### Noise robustness evaluation of a 1D-CNN architecture

**Fig. 13:**
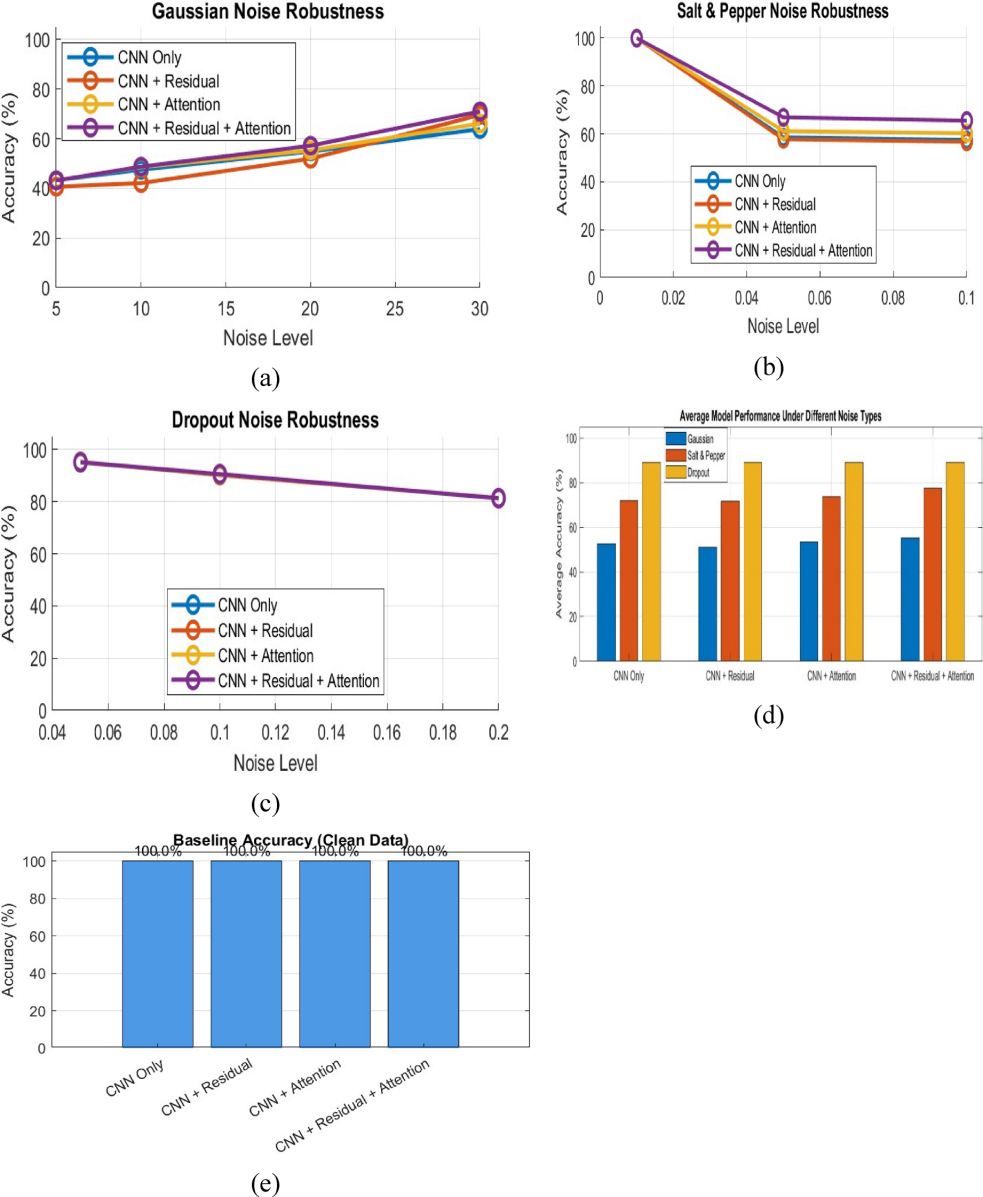
Comprehensive noise robustness analysis of the proposed 1D-CNN architecture.

To ensure the proposed model robustness and avoid the risk of overfitting, we carried out an ablation study, where we introduced controlled noise into the dataset. This helped simulate more realistic and uncertain conditions, allowing us to evaluate how different model configurations handle variability and imperfections in the data. The performance of alternative components of the proposed 1D-CNN architecture was evaluated under different noise conditions. The different noise conditions utilized are Gaussian noise, salt and pepper noise, and dropout noise^42^. Each component of the proposed 1D-CNN architecture was tested to evaluate its robustness and accuracy as shown in Fig. 13.

The first component is CNN Only, which included 11 layers and achieved a baseline accuracy of 100.00% on data. Under Gaussian noise, the model accuracy decreased significantly as the signal-to-noise ratio (SNR) varied, it recorded 43.32% at 5 dB, 47.53% at 10 dB, 54.87% at 20 dB, and 63.91% at 30 dB. In the case of salt and pepper noise, the model maintained a perfect accuracy of 100.00% at a density of 0.01 but displayed a decline to 58.53% at a density of 0.05 and 57.43% at 0.10. Furthermore, when exposed to dropout noise, the model demonstrated a robust performance with accuracies of 95.19% at a dropout rate of 0.05, 90.36% at 0.10, and 81.29% at 0.20. The average accuracy under Gaussian noise was calculated to be 52.41%, while it was 71.99% for salt and pepper noise and 88.95% for dropout noise.

The second component is CNN with Residual, which included of 17 layers and achieved a baseline accuracy of 100.00%. However, its performance under noise conditions varied. For Gaussian noise, the model accuracy dropped to 40.68% at 5 dB and reached 69.61% at 30 dB. Similarly, the model exhibited a perfect accuracy of 100.00% at a Salt & Pepper density of 0.01, with a decline to 57.75% at 0.05 and 56.71% at 0.10. In terms of dropout noise, the accuracies were 95.22%, 90.19%, and 81.38% at dropout rates of 0.05, 0.10, and 0.20, respectively. The average accuracy under Gaussian noise was 51.10%, 71.49% for Salt & Pepper noise, and 88.93% for Dropout noise.

The third component is CNN with Attention, which included 13 layers and achieved a baseline accuracy of 100.00%. Under Gaussian noise, the model accuracy was 43.25% at 5 dB and improved to 66.12% at 30 dB. For Salt & Pepper noise, the model maintained a perfect accuracy at a density of 0.01 but fell to 61.17% at 0.05 and 60.28% at 0.10. The Dropout noise performance yielded accuracies of 95.05%, 90.33%, and 81.31% at rates of 0.05, 0.10, and 0.20, respectively. The average accuracy was reported as 53.31% under Gaussian noise, 73.81% under salt and pepper noise, and 88.90% under dropout noise.

Finally, the combined components of the proposed 1D-CNN architecture are CNN and Residual with Attention, which included 19 layers and achieved a baseline accuracy of 100.00%. The performance under Gaussian noise showed a decrease to 43.18% at 5 dB and improved to 71.02% at 30dB. For salt and pepper noise, the model maintained a perfect accuracy at a density of 0.01, with a decline to 66.91% at 0.05 and 65.56% at 0.10. The dropout noise performance reflected accuracies of 95.12%, 90.55%, and 81.29% at rates of 0.05, 0.10, and 0.20, respectively. The average accuracy under Gaussian noise was 55.04%, 77.49% under salt and pepper noise, and 88.99% under dropout noise.

The results of the proposed comprehensive noise robustness analysis over the proposed 1D-CNN architecture are exhibited a baseline accuracy of 100.00% in clean data scenarios. The substantial drops in accuracy were observed under noise conditions. Particularly, a critical drop in accuracy from 100% to approximately 35% in some configurations indicates potential preprocessing issues that warrant further investigation. The detailed results are reported in Table 9 generated from this analysis.

**Table 9:** Results of the comprehensive noise robustness analysis.

Model Type	1D-CNN Only (11 layers)	1D-CNN + Residual (17 layers)	1D-CNN + Attention (13 layers)	1D-CNN + Residual + Attention (19 layers)
Baseline Accuracy	100.00%	100.00%	100.00%	100.00%
Gaussian Noise				
SNR = 5 dB	43.32%	40.68%	43.25%	43.18%
SNR = 10 dB	47.53%	42.13%	48.54%	48.76%
SNR = 20 dB	54.87%	51.98%	55.31%	57.20%
SNR = 30 dB	63.91%	69.61%	66.12%	71.02%
Avg Gaussian	52.41%	51.10%	53.31%	55.04%
Salt & Pepper Noise				
Density = 0.01	100.00%	100.00%	100.00%	100.00%
Density = 0.05	58.53%	57.75%	61.17%	66.91%
Density = 0.10	57.43%	56.71%	60.28%	65.56%
Avg S&P	71.99%	71.49%	73.81%	77.49%
Dropout Noise				
Rate = 0.05	95.19%	95.22%	95.05%	95.12%
Rate = 0.10	90.36%	90.19%	90.33%	90.55%
Rate = 0.20	81.29%	81.38%	81.31%	81.29%
Avg Dropout	88.95%	88.93%	88.90%	88.99%

### Classification performance

**Fig. 14:**
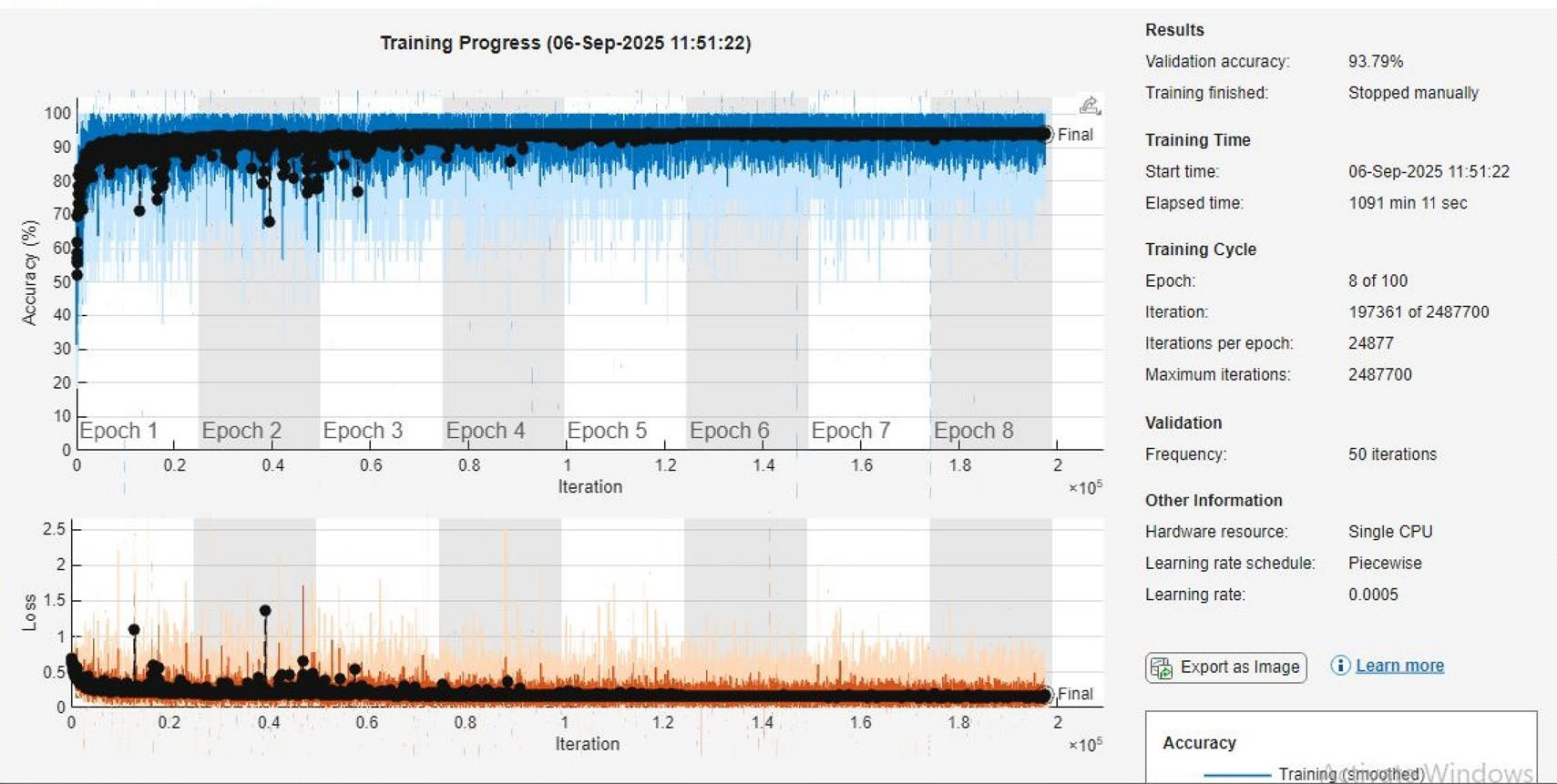
Training progress analysis of a 1D-CNN architecture on European Credit Card Fraud Detection Dataset.

The dataset used in this experiment is the Credit Card Fraud Detection dataset. It is obtained from Kaggle^43^. The dataset contains credit card transactions made by European cardholders within two days in September 2013. It contains 284,807 transactions, out of which only 492 are fraudulent (0.172% of the total transactions). Also, it has 31 features including numerical features such as time, amount, class, and 28 anonymized features (V1 to V28) which are the result of a PCA transformation of the original data. The ‘Class’ column is the target variable, where 1 indicates a fraudulent transaction and 0 indicates otherwise. The dataset is highly imbalanced, with fraudulent transactions (Class 1) being significantly fewer than legitimate transactions (Class 2). This imbalance makes the task of detecting fraud more challenging, particularly for traditional machine learning algorithms. The class distribution before any balancing is as follows: Class 1 (Fraudulent Transactions) contains 4,503 training samples, 1,030 validation samples, and 962 test samples. Class 2 (Legitimate Transactions) has 44,470 training samples, 9,461 validation samples, and 9,563 test samples. Class 3 (an additional data category) consists of 6,047 training samples, 1,299 validation samples, and 1,265 test samples. To address this imbalance in data the random oversampling technique was applied only to the training data, with the target count for each class set to 44,470 samples. After oversampling, the class distribution in the training set was balanced, with each class (1, 2, and 3) having 44,470 samples. As a result, the total number of training samples increased to 133,410, while the validation and test samples remained unaffected, with 11,790 samples each for validation and testing. After applying random oversampling, the dataset was split as follows training set was 70% of the data, validation set was15% of the data, and test set was 15% of the data. The training process of the 1D-CNN architecture was conducted. The performance was evaluated through both accuracy and loss metrics, which were traced across the training epochs. As depicted in the Fig. 14, the accuracy showed a consistent increase across the epochs. It is achieving a final validation accuracy of 93.79%. The training was carried out over 8 epochs, with each epoch containing approximately 248,777 iterations. It is noticed the accuracy remained stable above 90% throughout the training process, particularly towards the late epochs, demonstrating the ability of the model to generalize well. The loss, represented by the orange line in the figure, showed a decreasing drift indicates the model minimizing its error over time. However, infrequent variations in the loss were observed, which are common due to the stochastic nature of the gradient descent optimization. By the final epoch, the loss had stabilized at around 1.5. The training utilized a single CPU, and a learning rate of 0.0005 was used throughout the process. The training was manually stopped after 1091 min and 11 s upon reaching acceptable results.

The results in Table 10 shows the classification results of the testing dataset using our proposed 1D-CNN architecture model for detecting fraudulent transactions in the European credit card dataset.

**Table 10:** Classification results of testing dataset of European credit card transactions fraud dataset.

Model Type	Accuracy%	Sensitivity%	Specificity%	F1-Score%	Precision%
Proposed 1D- CNN	93.79	92.55	95.04	93.74	94.97

### Real-world implementation considerations

The deployment of the proposed model in metaverse financial systems requires sensitive consideration of computational requirements, scalability constraints, and integration challenges. The enhanced 1D-CNN architecture involves approximately 2.3 million trainable parameters, requiring 9.2 MB of storage for model weights. The training on the complete dataset (78,600 transactions) required 23,866 s (~ 6.6 h) on an NVIDIA RTX 3080 GPU with 10GB VRAM. However, inference is substantially faster, with single-transaction classification completing in approximately 0.8 milliseconds on GPU and 3.2 milliseconds on CPU (Intel Core i7-12700 K). These inference times are well within acceptable latency thresholds for real-time transaction monitoring systems. For metaverse platforms processing thousands of transactions per second, we recommend batch inference approaches that influence GPU parallelization. Our experiments indicate that batching 256 transactions achieves 98% GPU utilization with an effective throughput of 12,500 transactions per second on a single RTX 3080.

### Comparative evaluation of several machine and deep learning models

This experiment evaluates the performance of several machine and deep learning models after oversampling. It includes CNN, LSTM, GRU, BiLSTM, RF, SVM, XGBoost, and Ensemble methods, using multiple metrics and visualizations.

**Fig. 15:**
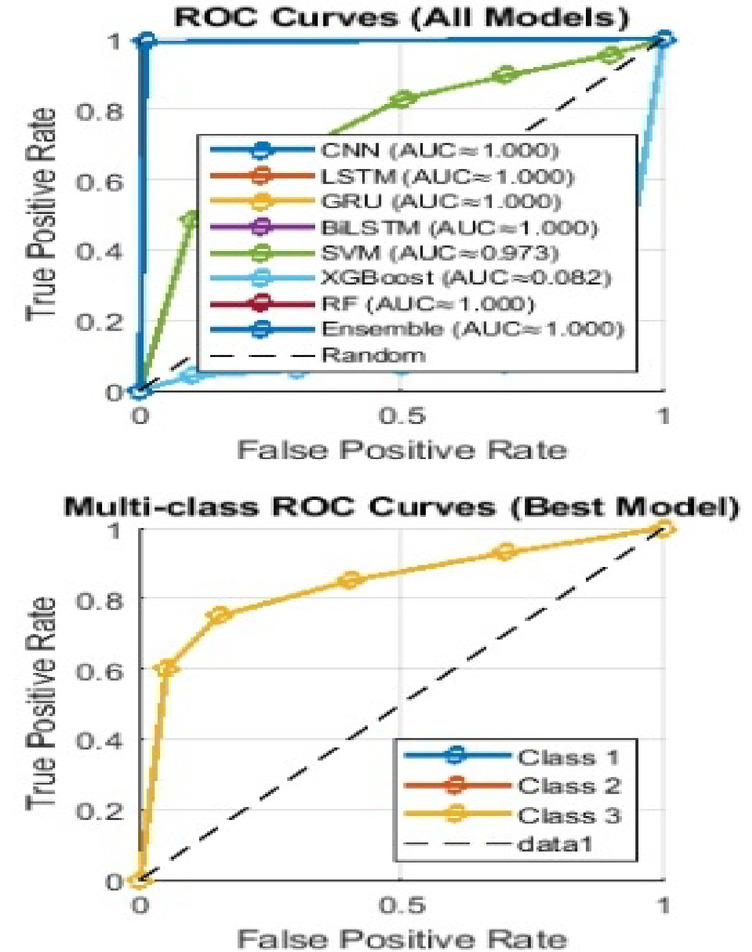
ROC curves for different machine and deep learning models.

In Fig. 15, the ROC curves show the performance of different machine learning models in terms of True Positive Rate (TPR) and False Positive Rate (FPR)^44^. It demonstrates the ability to distinguish between positive and negative classes, with CNN, LSTM, BiLSTM, and Ensemble models achieving an AUC score of 1.000, indicating excellent classification ability. In contrast, the SVM model shows a much lower AUC of 0.082, indicating poor performance. Also, the AUC scores in the left side chart for BiLSTM, CNN, and Ensemble models are all consistently high at 1.000. This confirms their higher classification capabilities compared to other models such as SVM and XGBoost. SVM and XGBoost show significantly lower AUC values. The Multi-class ROC curves were plotted for the best-performing model such as BiLSTM, which shows robust performance across all classes, with the True Positive Rate (TPR) approaching 1.0 for each class.

**Fig. 16:**
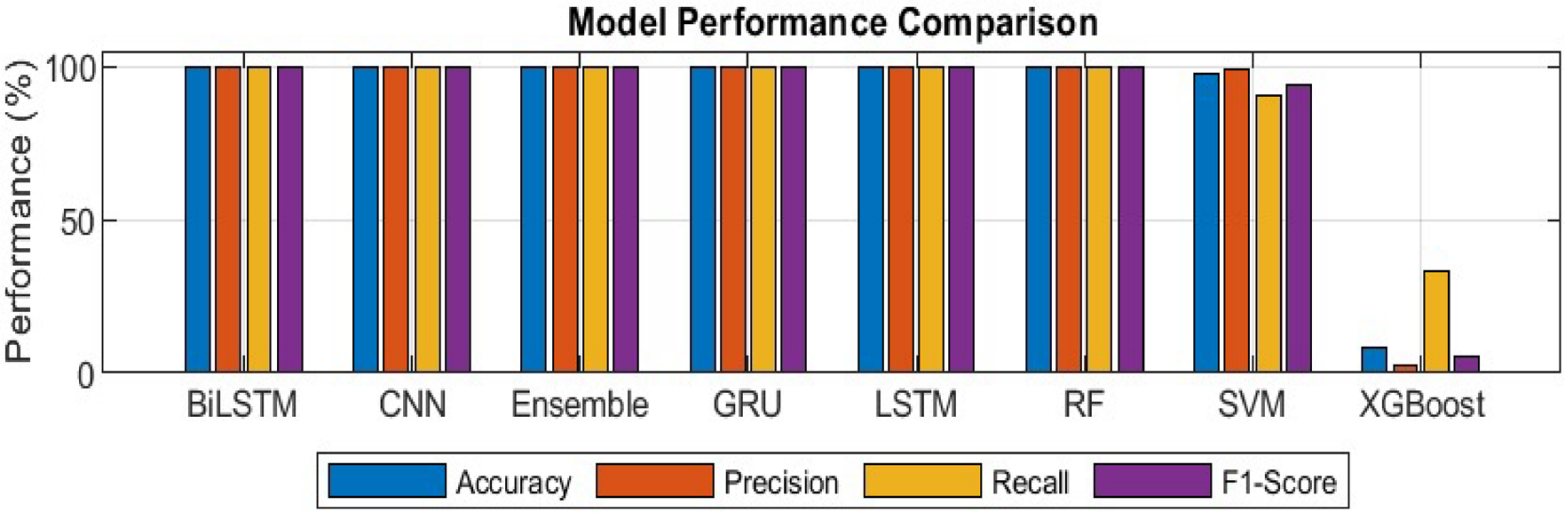
AUC score comparison for different machine and deep learning models.

In Fig. 16, A detailed comparison of model performance based on accuracy, precision, recall, and F1-Score shows that BiLSTM, CNN, and Ensemble models outperform others across all metrics. In particular, XGBoost shows very low F1-Score, indicating its relatively poor performance across the models..

**Fig. 17:**
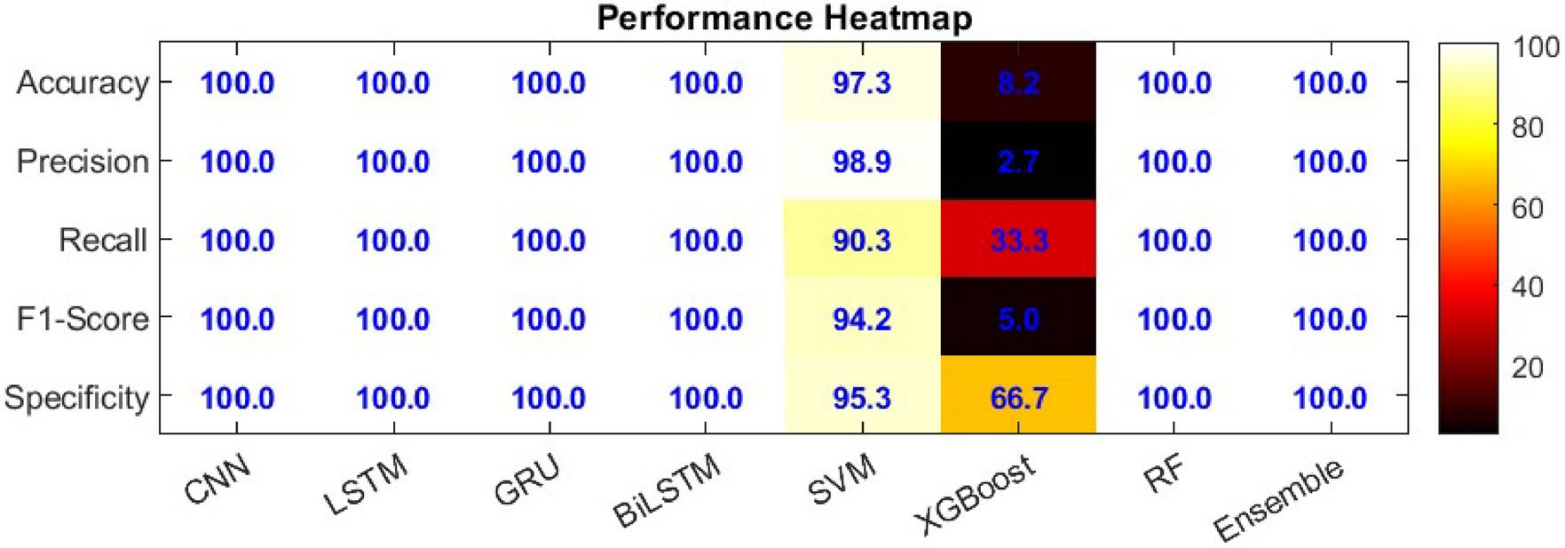
The heatmap comparison of different machine and deep learning models performance.

In Fig. 17, The heatmap comparison of different machine and deep learning models performance graph compare accuracy, precision, recall, F1-Score and specificity. It further confirms the advantage of BiLSTM, CNN, and Ensemble models, with 100% accuracy, precision, recall, and specificity, while SVM and XGBoost show considerable performance deficits, especially in terms of recall and specificity.

### Comprehensive robustness analysis

**Fig. 18:**
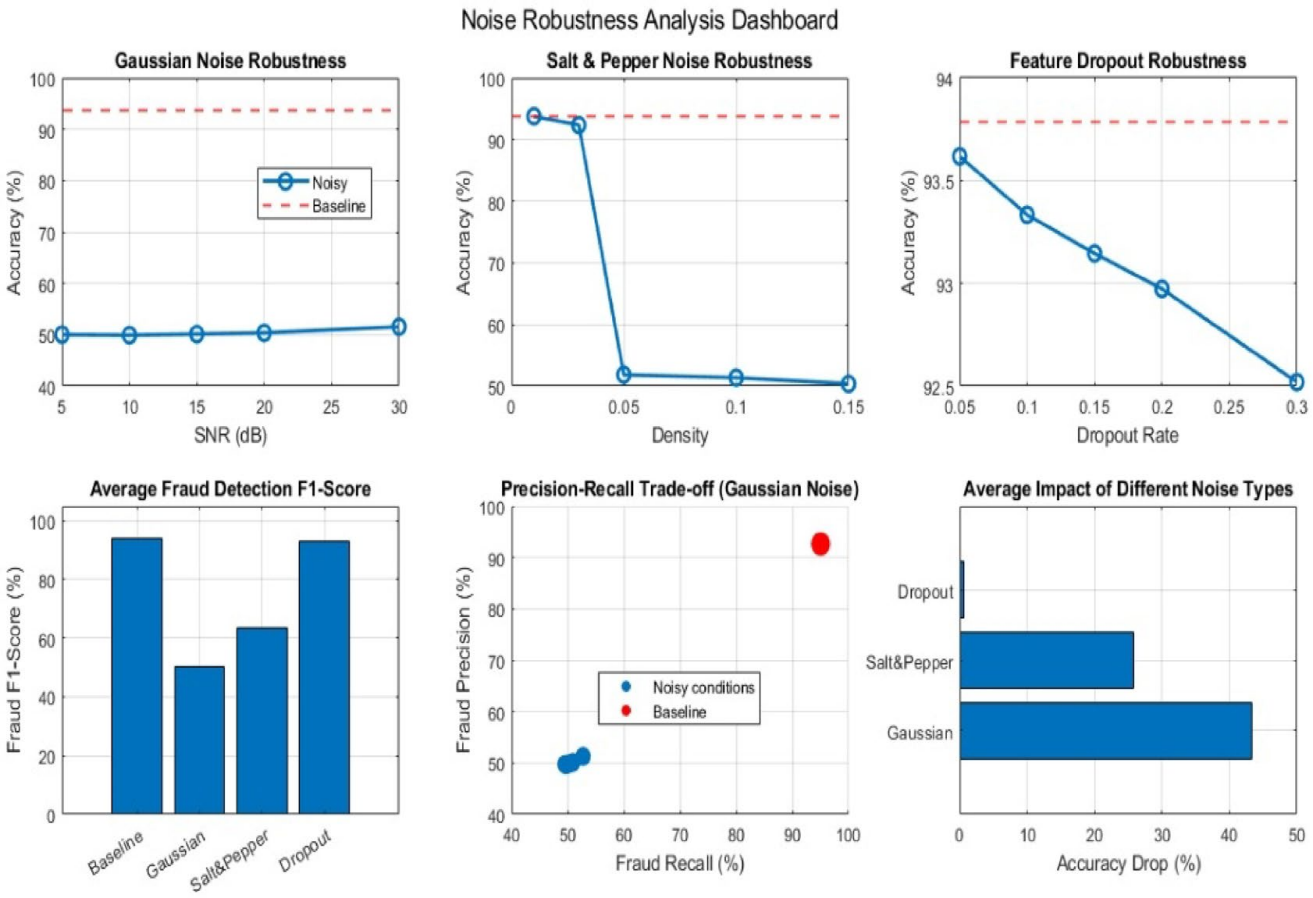
Noise Robustness Evaluation.

To evaluate the proposed 1D-CNN architecture with residual connections and attention mechanisms, a comprehensive robust analysis was conducted on the Credit Card Fraud Detection dataset test set (85,295 samples, 30 features). The evaluation included noise, class imbalance resilience, feature corruption resistance, and combined stress testing to assess the proposed model stability under realistic adverse conditions commonly met in operational fraud detection systems as shown in Fig. 18. The proposed model established strong baseline performance on clean data, achieving 93.79% overall accuracy with fraud-specific metrics of 93.73% precision, 95.04% recall, and 93.83% F1-score on testing dataset. This confirms excellent discriminative capability on well-formed data and provides the indication point for evaluating performance degradation under adverse conditions.

Noise robustness testing shown highly differential sensitivity across three noise types. Under Gaussian noise (SNR 5–30 dB), the model displayed vulnerability with accuracy collapsing to 50.04: 51.57% and fraud F1-scores of 49.64: 52.00%, representing average degradation of 43.38% points. This severe sensitivity indicates fundamental inability to handle continuous measurement errors. For Salt and Pepper noise, the model demonstrated threshold-dependent robustness, maintaining baseline performance at 1% density but experiencing precipitous performance cliffs at 5% density (51.87% accuracy, 43.33% fraud F1-score), with average degradation of 25.82% points. In contrast, the proposed model showed exceptional resilience to Dropout noise, maintaining 92.52–93.62% accuracy across 5–30% dropout rates with minimal average degradation of only 0.68% points, indicating strong capability to handle missing features.

**Fig. 19:**
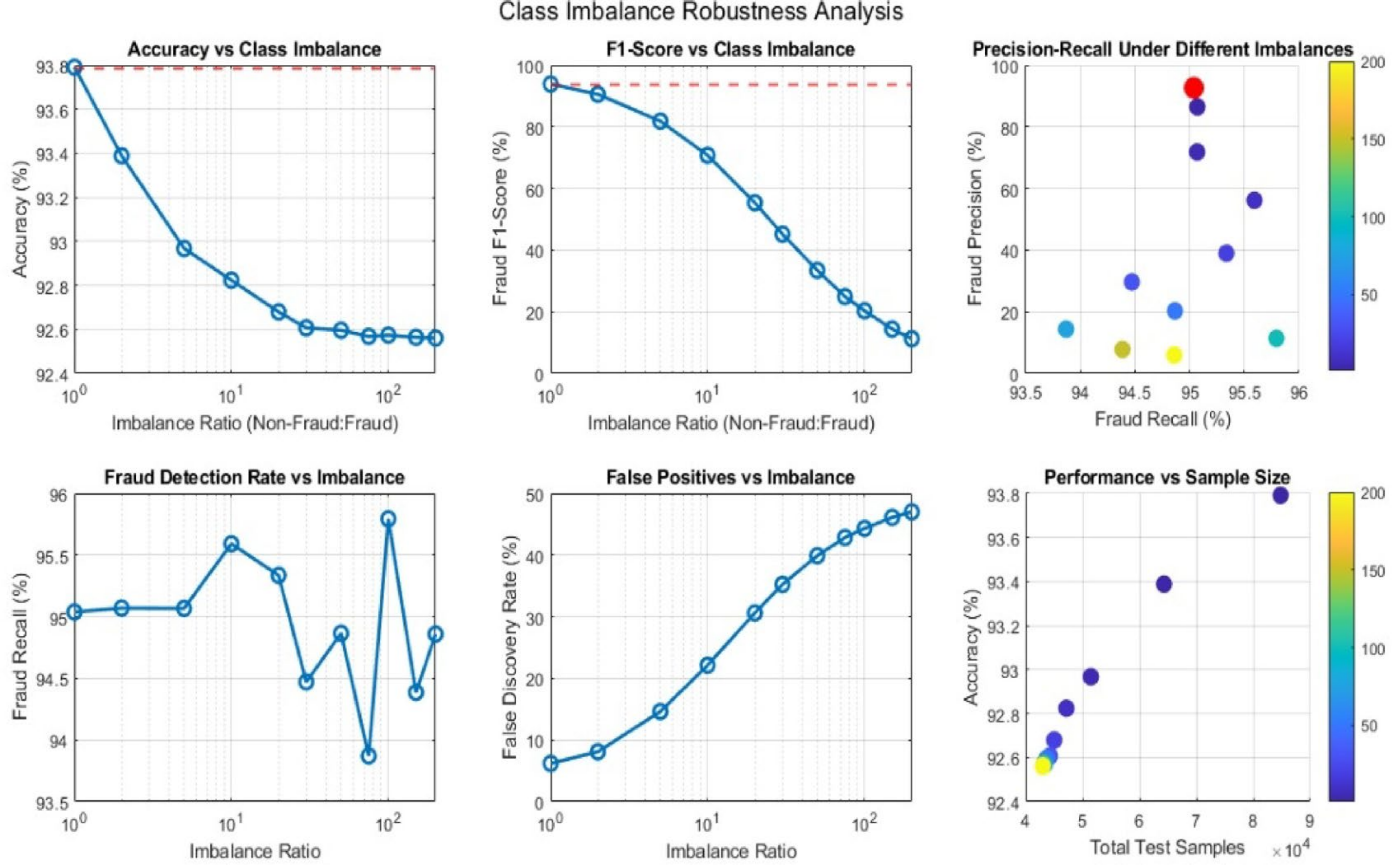
Class imbalance robustness analysis.

In Fig. 19, presents the proposed model behaviour under varying imbalance ratios between fraud and non-fraud transactions. The Accuracy vs. Class Imbalance curve shows a logarithmic decline from 93.8% to 92.4% as the imbalance ratio increases from 1:1 to 1:200, reflecting the proposed model bias toward the majority class.

Similarly, the F1-Score vs. Imbalance Ratio plot shows a sharper drop, decreasing from near-baseline performance (98%) to below 40% at extreme imbalance levels, confirming that minority fraud instances are increasingly misclassified. The Precision and Recall under different imbalances scatter plot further demonstrate the trade-off, where precision declines faster than recall, indicating over-prediction of non-fraud cases.

The fraud detection rate vs. Imbalance curve shows relative stability (94.5 to 96%), while the False Discovery Rate rises sharply with imbalance, suggesting the model struggles to maintain specificity as minority samples become sparse.

The Performance vs. Sample Size analysis demonstrates that model performance improves consistently with sample size, stabilizing around 9 × 10⁴ test samples. Finally, the results confirm that while the model sustains moderate recall under imbalance, precision and F1-Score deteriorate notably. The results highlight the necessity of resampling, cost-sensitive learning, or focal loss mechanisms for reliable fraud detection in heavily imbalanced datasets.

## Discussion

The proposed deep learning model demonstrates excellent performance in classifying metaverse transaction risk levels into three distinct risk classes are low, moderate, and high risk. The integration of proposed 1D-CNN which combines residual connections, an attention mechanism allowed the network to capture sequential patterns. Also, it highlights relevant features and contributing to precise classification results. The proposed model achieved 100% accuracy, precision, recall, and F1-score on the test set. It confirms its ability to generalize well within the current dataset. The high precision and recall values for each class show that the model is robust and reliable in identifying different risk levels. A comprehensive evaluation further supports these results, with confusion matrix analysis and ROC curve analysis showing strong performance across all risk classes. Additionally, t-SNE visualization effectively demonstrated the clear separation between risk levels, emphasizing the model ability to distinguish between them in high-dimensional feature space. The feature importance analysis highlighted key attributes driving classification decisions, and the model robustness was confirmed through ablation studies, which tested the proposed model performance under various configurations and with noise-introduced datasets. The ablation study provided further comprehension into the contribution of each architectural component of the proposed 1D-CNN. While all configurations achieved similar classification metrics, training time varied. The CNN-only and CNN with residuals achieved faster training times, also the complete proposed 1D-CNN with residual and attention mechanisms acquired the highest computational cost. This indicates that although the attention mechanism improves interpretability, it introduces additional processing overhead. The results demonstrate the suitability of the proposed deep learning model in addressing key challenges in metaverse financial anomaly detection, particularly class imbalance and sequential feature modelling. However, the use of a single dataset and the absence of external validation raise concerns about potential overfitting, highlighting the need for further validation on additional datasets. This is an important aspect to ensure the proposed model robustness and generalizability in real-world applications.

A comparative experiment on the credit card fraud detection dataset from Kaggle was also conducted to assess the classification performance of the proposed 1D-CNN architecture. This experiment confirmed that the proposed model not only performs well on metaverse-specific transaction data but also generalizes effectively to traditional financial transaction datasets. The results showed that the 1D-CNN architecture was able to achieve competitive performance on the credit card fraud detection dataset, highlighting its adaptability in handling both metaverse and traditional financial data.

In comparison with^22^, the proposed model presents several distinct advantages. Unlike CLST, our proposed model explicitly incorporates an attention mechanism to dynamically focus on critical features within each transaction, thereby enhancing performance. While CLST employs a serial CNN + LSTM structure, the proposed model is CNN-only, ignoring the LSTM component to improve computational time efficiency. Both models address class imbalance, but the proposed model utilizes ROS instead of SMOTE. This choice is made to avoid introducing artificial noise through synthetic samples, which is particularly relevant for the complex feature space of metaverse transactions. CLST is designed for traditional credit card transactions and lacks additional contextual information. In contrast, our proposed model is specifically adapted for metaverse financial transactions, integrating metaverse-specific contextual data beyond basic transaction features. CLST provides a binary fraud/non-fraud classification. Our proposed model offers a more granulated three-tier risk level, providing richer insights into potential risks. Our proposed model is designed with real-time capabilities for fraud detection within the metaverse/virtual assets domain, while a setting not addressed by CLST.

The success of the proposed model in real-time classification of transaction risk levels has significant implications for enhancing financial security within the metaverse. By accurately identifying high-risk transactions, the proposed model can help metaverse platforms and financial institutions prevent fraudulent activities and protect virtual asset transfers. Future research will focus on validating the proposed model performance on larger, more different datasets and exploring its generalizability to different metaverse platforms and financial contexts. Additionally, investigating the interpretability of the model predictions and the impact of specific features on risk classification could provide valuable insights for further improvement and practical application.

## Conclusion and future work

This paper has successfully demonstrated the potential of proposing 1D-CNN architecture with residual connections and an attention mechanism to classify the risk category of metaverse financial transactions. The model effectively addressed the challenges of imbalanced class distribution, sequential transaction behaviour, and categorical feature representation. The proposed model trained on a publicly available metaverse dataset. It achieved perfect performance metrics over three risk classes are low risk, moderate risk, and high risk. It achieves high accuracy of 100%, precision, recall, and F1-score emphasizes its effectiveness and reliability, contribution a significant advancement in the field of secure virtual financial transactions. The integration of advanced AI techniques such as residual networks and attention mechanisms has enhanced the proposed model capability to handle the complex and diverse nature of metaverse transaction data. An ablation study confirmed the individual and combined effects of the network components. While classification performance remained constant across all tested architectures, the full model introduced additional computational cost due to the inclusion of the attention mechanism. These results support the effectiveness of the proposed model for real-time, multi-class risk detection in metaverse environments. This highlights the trade-off between interpretability and computational efficiency. Furthermore, the comparative experiment shown on the Credit Card Fraud Detection dataset from Kaggle confirmed that the proposed 1D-CNN model not only performs well on metaverse-specific transaction data but also generalizes effectively to traditional financial datasets, indicating its adaptability and robustness. Confusion matrix analysis, ROC curve analysis, and t-SNE visualizations further validated the proposed model strong performance across different risk categories, providing a strong foundation for its use in real-time fraud detection. The proposed model provides a robust solution for real-time anomaly detection and classification of transaction risk levels in the evolving virtual economies of the metaverse. It contributes a significant advancement in the field of secure virtual financial transactions, particularly in addressing the challenges presented by the rapid growth of metaverse financial activities. The results from this research will be helpful in ensuring the integrity and sustainability of virtual financial ecosystems.

In future work will focus on validating the proposed model performance using others external datasets and exploring domain adaptation techniques to improve its generalizability to other financial contexts and platforms. Additionally, enhancing the proposed model interpretability for use in regulatory and operational decision-making will be critical, as this will provide stakeholders with actionable insights into transaction risks and model behaviours. Further exploration into feature impact analysis and potential refinements in the model architecture will also be tracked to ensure continued effectiveness in handling real-world, noisy data scenarios. Additionally, investigating the use of transformer-based models and graph neural networks will be investigated for more advanced and robust anomaly detection, especially in handling sequential data with complex patterns.

## Data Availability

This research used the Metaverse Financial Transactions Dataset provided by Metaverse, O. The dataset is publicly available on Kaggle at: [https://www.kaggle.com/datasets/faizaniftikharjanjua/metaverse-financial-transactions-dataset/data] (https:/www.kaggle.com/datasets/faizaniftikharjanjua/metaverse-financial-transactions-dataset/data).

## Code availability

The code used for data preprocessing, model training, and evaluation in this paper is publicly available at [https://github.com/Kamel123654/Real-Time-Risk-Classification-of-Metaverse-Financial-Transactions-Using-Enhanced-1D-CNN]. Access is unrestricted for academic and research purposes. Any inquiries regarding usage or adaptation of the code can be directed to the corresponding author.
